# Genetics of Smoking and Risk of Atherosclerotic Cardiovascular Diseases: A Mendelian Randomization Study

**DOI:** 10.1001/jamanetworkopen.2020.34461

**Published:** 2021-01-19

**Authors:** Michael G. Levin, Derek Klarin, Themistocles L. Assimes, Matthew S. Freiberg, Erik Ingelsson, Julie Lynch, Pradeep Natarajan, Christopher O’Donnell, Daniel J. Rader, Philip S. Tsao, Kyong-Mi Chang, Benjamin F. Voight, Scott M. Damrauer

**Affiliations:** 1 Division of Cardiovascular Medicine, University of Pennsylvania Perelman School of Medicine, Philadelphia; 2 Department of Medicine, University of Pennsylvania Perelman School of Medicine, Philadelphia; 3 Corporal Michael J. Crescenz VA Medical Center, Philadelphia, Pennsylvania; 4 Malcolm Randall VA Medical Center, Gainesville, Florida; 5 Department of Surgery, University of Florida, Gainesville; 6 Palo Alto VA Healthcare System, Palo Alto, California; 7 Division of Cardiovascular Medicine, Department of Medicine, Stanford University School of Medicine, Stanford, California; 8 Stanford Cardiovascular Institute, Stanford University, Stanford, California; 9 Division of Cardiovascular Medicine, Vanderbilt University Medical Center, Nashville, Tennessee; 10 Geriatric Research Education and Clinical Centers, Veterans Affairs Tennessee Valley Healthcare System, Nashville; 11 Department of Medicine, Vanderbilt University Medical Center, Nashville, Tennessee; 12 Stanford Diabetes Research Center, Stanford University, Stanford, California; 13 Now with GlaxoSmithKline, San Francisco, California; 14 Edith Nourse VA Medical Center, Bedford, Massachusetts; 15 VA Informatics and Computing Infrastructure, Salt Lake City, Utah; 16 Cardiovascular Research Center, Massachusetts General Hospital, Boston; 17 Broad Institute of Harvard and MIT, Cambridge, Massachusetts; 18 Department of Medicine, Harvard Medical School, Boston, Massachusetts; 19 VA Boston Healthcare System, Boston, Massachusetts; 20 Department of Genetics, University of Pennsylvania Perelman School of Medicine, Philadelphia; 21 Institute for Translational Medicine and Therapeutics, University of Pennsylvania Perelman School of Medicine, Philadelphia; 22 Stanford Cardiovascular Institute, Division of Cardiovascular Medicine, Department of Medicine, Stanford University, Palo Alto, California; 23 Department of Systems Pharmacology and Translational Therapeutics, University of Pennsylvania Perelman School of Medicine, Philadelphia; 24 Department of Surgery, University of Pennsylvania Perelman School of Medicine, Philadelphia

## Abstract

**Importance:**

Smoking is associated with atherosclerotic cardiovascular disease, but the relative contribution to each subtype (coronary artery disease [CAD], peripheral artery disease [PAD], and large-artery stroke) remains less well understood.

**Objective:**

To determine the association between genetic liability to smoking and risk of CAD, PAD, and large-artery stroke.

**Design, Setting, and Participants:**

Mendelian randomization study using summary statistics from genome-wide associations of smoking (UK Biobank; up to 462 690 individuals), CAD (Coronary Artery Disease Genome Wide Replication and Meta-analysis plus the Coronary Artery Disease Genetics Consortium; up to 60 801 cases, 123 504 controls), PAD (VA Million Veteran Program; up to 24 009 cases, 150 983 controls), and large-artery stroke (MEGASTROKE; up to 4373 cases, 406 111 controls). This study was conducted using summary statistic data from large, previously described cohorts. Review of those publications does not reveal the total recruitment dates for those cohorts. Data analyses were conducted from August 2019 to June 2020.

**Exposures:**

Genetic liability to smoking (as proxied by genetic variants associated with lifetime smoking index).

**Main Outcomes and Measures:**

Risk (odds ratios [ORs]) of CAD, PAD, and large-artery stroke.

**Results:**

Genetic liability to smoking was associated with increased risk of PAD (OR, 2.13; 95% CI, 1.78-2.56; *P* = 3.6 × 10^−16^), CAD (OR, 1.48; 95% CI, 1.25-1.75; *P* = 4.4 × 10^−6^), and stroke (OR, 1.40; 95% CI, 1.02-1.92; *P* = .04). Genetic liability to smoking was associated with greater risk of PAD than risk of large-artery stroke (ratio of ORs, 1.52; 95% CI, 1.05-2.19; *P* = .02) or CAD (ratio of ORs, 1.44; 95% CI, 1.12-1.84; *P* = .004). The association between genetic liability to smoking and atherosclerotic cardiovascular diseases remained independent from the effects of smoking on traditional cardiovascular risk factors.

**Conclusions and Relevance:**

In this mendelian randomization analysis of data from large studies of atherosclerotic cardiovascular diseases, genetic liability to smoking was a strong risk factor for CAD, PAD, and stroke, although the estimated association was strongest between smoking and PAD. The association between smoking and atherosclerotic cardiovascular disease was independent of traditional cardiovascular risk factors.

## Key Points

**Question:**

Are there differential associations between genetic liability to smoking and atherosclerotic cardiovascular disease (ASCVD) outcomes (coronary artery disease, peripheral artery disease, and ischemic stroke)?

**Findings:**

In this mendelian randomization study including summary data for more than 1 million individuals, genetic liability to smoking was associated with increased risk of ASCVD, with the largest association with peripheral artery disease, independent from other cardiovascular risk factors.

**Meaning:**

Findings of this study indicate that genetic liability to smoking has a strong, independent effect on ASCVD but is most strongly associated with peripheral artery disease; further studies of the differential effects of other ASCVD risk factors may improve risk stratification and treatment.

## Teaser

This mendelian randomization study evaluates summary statistics from genome-wide associations involving more than 1 million individuals to assess whether there are differential associations between genetic liability to smoking and risk of coronary artery disease, peripheral artery disease, and large-artery stroke.

## Introduction

Atherosclerotic cardiovascular disease (ASCVD) can affect numerous vascular beds throughout the body, with clinical manifestations including coronary artery disease (CAD), stroke, and peripheral artery disease (PAD). Smoking tobacco is consistently among the leading risk factors for ASCVD; however, the relative contribution of smoking to the individual ASCVD outcomes remains less well studied. Observational studies have examined these ASCVD outcomes together, with a recent study by Ding et al finding the strongest association between smoking and incident PAD compared with CAD or stroke.^1,2,3,4^ Observational study designs may be limited, however, by modest overall sample size, measurement error, and risk of residual confounding.^5^

A number of studies during the last several decades have identified detrimental effects of smoking on traditional cardiovascular risk factors, including blood pressure, lipids, and diabetes.^6,7^ Smoking also has independent effects on inflammation, endothelial function, and platelet aggregation.^6^ Despite the clear observational links between smoking and atherosclerosis, whether the effect of smoking on ASCVD is primarily mediated through correlated alterations of traditional cardiovascular risk factors, or operates via independent mechanisms is less clear. Because the detrimental effects of smoking may persist for decades,^4^ clarifying the basis of the smoking-atherosclerosis relationship could enable more targeted risk-reduction strategies among both current and former smokers and identify novel treatment strategies for those at highest risk of ASCVD.

Recently, large genome-wide association studies (GWASs) of smoking, coronary artery disease, stroke, and peripheral artery disease have identified genetic loci associated with each of these conditions.^8,9,10,11^ The mendelian randomization (MR) framework leverages the natural randomization of genetic variation at conception to mitigate risks of confounding that limit other observational methods. Genetic variants are randomly allocated across the population at meiosis and conception, mimicking randomization in a clinical trial. Under certain assumptions, the MR framework mitigates risks from environmental confounding and reverse causality that may affect other observational methods.^12^ This approach may allow for more precise quantification of potential differences between exposure-outcome pairs.^12^ The method has further been extended to consider exposures jointly, a form of mediation analysis that enables the estimation of the direct effect of each exposure on an outcome of interest.^13^ In the present analysis, we studied the association between genetic liability to smoking (defined by genetic variants associated with measures of smoking) and ASCVD and cardiometabolic outcomes.

Here, leveraging population-scale human genetics data from GWASs, we used the MR framework with genetic variants as instrumental variables to (1) estimate the total effect sizes for associations between smoking and risk of PAD, CAD, and stroke, the primary manifestations of ASCVD; (2) validate the association between smoking and traditional cardiovascular and inflammatory risk factors for ASCVD; and (3) assess the extent to which traditional cardiovascular and inflammatory risk factors affect the relationship between smoking and ASCVD outcomes.

## Methods

### Smoking Genetic Instrument Selection

Genetic variants were used as instrumental variables for 2 different measures of smoking. These genetic exposures used as proxies for smoking are referred to herein as “genetic liability to smoking.” The 2 measures of smoking used throughout the study are lifetime smoking index^11^ and smoking initiation.^14^ The primary measure of smoking was lifetime smoking index, a previously validated continuous measure that accounts for self-reported smoking status, age at initiation, age at cessation, number of cigarettes smoked per day, and a simulated half-life constant that captures the decreasing effect of smoking on health outcomes following a given exposure (Table).^11^ A genetic instrument for lifetime smoking index was constructed from summary statistics of a GWAS of UK Biobank participants (UK Biobank; N = 462 690) using independent (*r*^2^ = 0.001; distance, 10 000 kilobases; European-ancestry participants of the 1000 genomes project) genome-wide significant variants (*P* < 5 × 10^−8^) as the exposure, as previously described.^11^ Each SD increase in the lifetime smoking index instrument corresponds to an individual smoking 20 cigarettes daily for 15 years and stopping 17 years ago, or smoking 60 cigarettes daily for 13 years and stopping 22 years ago. The smoking index instrument was previously validated using an independent set of participants and with positive-control outcomes of coronary artery disease and lung cancer.^11^ To further validate the instrument, we performed 2-sample MR using lifetime smoking index as the exposure, and smoking initiation, age of smoking initiation, smoking cessation, and cigarettes per day,^14^ as reported by GWAS and Sequencing Consortium of Alcohol and Nicotine, as outcomes. To evaluate the strength of the smoking index instrument, we calculated the *F* statistic.^15^ In sensitivity analyses, genetic liability to smoking was assessed using smoking initiation as the exposure, rather than lifetime smoking index. This study is reported according to the Strengthening the Reporting of Observational Studies in Epidemiology (STROBE) reporting guideline for observational studies.^16^ This study used only publicly available, deidentified summary statistics from previously published works, making it exempt from institutional review board review according to the University of Pennsylvania regulations.

**Table.** Overview of Genetic Data Sets^a^

Trait	Cohort	Year	PMID	No.		
				Sample size	Cases	Controls
Smoking (lifetime smoking index)	UKB	2019	31689377	462 690		
Coronary artery disease	CARDIoGRAMplusC4D	2015	26343387	184 305	60 801	123 504
Peripheral artery disease	MVP	2019	31285632	174 992	24 009	150 983
Large-artery stroke	MEGASTROKE	2018	29531354	150 765	4373	406 111
Overweight	GIANT	2013	23563607	158 855	93 015	65 840
Diabetes	DIAGRAM+GERA+UKB	2018	30054458	655 666	61 714	1178
Hyperlipidemia	UKB	2018	29846171	462 933	56 753	406 180
Chronic kidney disease	CKDGen	2015	26831199	117 165	12 385	104 780
Hypertension	UKB	2018	29846171	462 933	2095	460 838
Body mass index	GIANT	2015	25673413	339 224	NA	NA
C-reactive protein	INTERVAL	2018	29875488	3301	NA	NA
Fasting glucose	MAGIC	2012	22581228	58 074	NA	NA
Fasting insulin	MAGIC	2012	22581228	51 750	NA	NA
HbA_1C_	MAGIC	2010	20858683	46 368	NA	NA
HDL cholesterol	GLGC	2013	24097068	187 167	NA	NA
Interleukin 6 receptor	INTERVAL	2018	28369058	3394	NA	NA
Interleukin 1β	INTERVAL	2018	29875488	3301	NA	NA
Interleukin 6	INTERVAL	2018	29875488	3301	NA	NA
LDL cholesterol	GLGC	2013	24097068	173 082	NA	NA
Serum creatinine (eGFRcrea)	CKDGen	2015	26831199	133 814	NA	NA
Systolic blood pressure	UKB	2018	29846171	436 419	NA	NA
Total cholesterol	GLGC	2013	24097068	187 365	NA	NA
Triglycerides	GLGC	2013	24097068	177 861	NA	NA
Waist circumference	GIANT	2015	25673412	232 101	NA	NA
Waist to hip ratio	GIANT	2015	25673412	224 459	NA	NA

Abbreviations: CARDIoGRAMplusC4D, Coronary Artery Disease Genome Wide Replication and Meta-analysis plus the Coronary Artery Disease Genetics Consortium; CKDGen, Chronic Kidney Disease Genetics Consortium; DIAGRAM, Diabetes Genetics Replication and Meta-analysis consortium; eGFRcrea, estimated glomerular filtration rate (creatinine); GERA, Genetic Epidemiology Research on Aging; GIANT, Genetic Investigation of Anthropometric Traits consortium; GLGC, Global Lipid Genetics Consortium; HbA_1C_, hemoglobin A_1C_; HDL, high-density lipoprotein; LDL, low-density lipoprotein; MAGIC, Meta-Analyses of Glucose and Insulin-Related Traits Consortium; MVP, Million Veteran Program; NA, not applicable; PMID, PubMed identification number; UKB, UK Biobank.

^a^ Overview of genetic data sets used in the mendelian randomization analyses. The number of cases and controls is reported for case-control studies, with total sample size reported for all studies.

### ASCVD Outcome Selection

The ASCVD outcomes were obtained from large GWASs of CAD, PAD, and large-artery stroke (Table). The CAD effects were obtained from the Coronary Artery Disease Genome Wide Replication and Meta-analysis plus the Coronary Artery Disease Genetics Consortium 1000 Genomes GWAS, which included 60 801 CAD cases and 123 504 controls of primarily European ancestry (77%) across 48 studies, including a combination of incident and prevalent disease.^8^ The PAD effects were obtained from the Million Veterans Program GWAS, which included 24 009 prevalent PAD cases and 150 983 controls of European ancestry enrolled at 63 Veterans Affairs (VA) Medical Centers across the United States.^9^ The large-artery stroke effects were obtained from the MEGASTROKE consortium GWAS, which included 4373 cases and 406 111 controls of European ancestry enrolled across 10 studies, including a combination of incident and prevalent cases.^10^

### Cardiometabolic Risk Factor Selection

The effects of genetic variants on cardiometabolic risk factors were obtained from publicly available summary statistics from GWASs of continuous traits (total cholesterol level, low-density lipoprotein cholesterol level, high-density lipoprotein cholesterol level, level of triglycerides, body mass index, waist to hip ratio, fasting glucose level, fasting insulin level, systolic blood pressure, estimated glomerular filtration rate, circulating C-reactive protein level, circulating interleukin 1B level, circulating interleukin 6 level, and circulating interleukin 6R level), and binary traits (type 2 diabetes, hypertension, hyperlipidemia, chronic kidney disease, and overweight) identified using the MR-Base platform (Table).^17,18,19,20,21,22,23,24^ These risk factors were selected as common markers of cardiometabolic risk, inclusion in risk calculators, and genetic, observational, and randomized clinical trial evidence of association with coronary artery disease.^25,26,27,28,29^

### Mendelian Randomization

**Figure 1.**
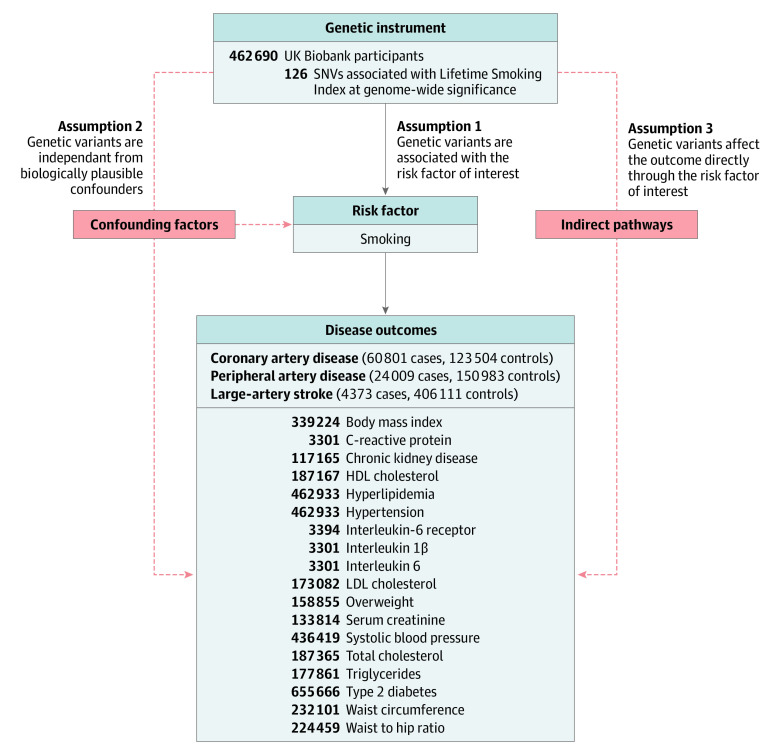
Mendelian Randomization Analysis Overview Overview of mendelian randomization analyses and major assumptions. Solid lines represent the direct pathway in which genetic variants serve as instruments for a risk factor of interest, and the effect on a disease outcome is measured. Dashed red lines represent pathways that potentially violate mendelian randomization assumptions. For atherosclerotic cardiovascular disease outcomes, the number of cases and controls are listed. For cardiometabolic outcomes, the total sample size is listed. See Table for additional cohort information. HDL indicates high-density lipoprotein; LDL, low-density lipoprotein; and SNV, single-nucleotide variant.

In the primary analysis, the total effect of lifetime smoking index on ASCVD outcomes (CAD, PAD, and stroke) was estimated using random-effects inverse-variance–weighted MR within the TwoSampleMR package in R (Figure 1).^5,30^ In sensitivity analyses, fixed-effects inverse-variance–weighted, maximum likelihood, weighted-median, penalized weighted-median, and MR pleiotropy residual sum and outlier methods were performed to account for potential violations of the instrumental variable assumptions, presence of horizontal pleiotropy, heterogeneity, and error in the instrument-exposure associations.^5,31,32^ The latter method (1) tests for the presence of horizontal pleiotropy, (2) removes pleiotropic genetic variants, and (3) tests for differences in estimates before and after outlier removal.^33^ Diagnostic leave-one-out, single single-nucleotide variant, and funnel-plot analyses were performed to visually assess for outliers and bias. The Egger bias intercept test was used to quantitatively detect evidence of horizontal pleiotropy. In sensitivity analysis, a genetic instrument for smoking initiation was used as the exposure.^14^ Because 2-sample MR of binary exposures provides effect estimates per 1-unit change in the exposure, the results of the effect of the smoking initiation exposure on ASCVD outcomes are expressed as odds of the outcome per 2.72-fold (1 log odds unit) increase in the odds of ever smoking.^34^ Differences in the effect of smoking on ASCVD outcomes in each vascular bed were estimated using the ratio of the odds ratios (ORs), based on a null hypothesis that the ORs for the associations between smoking and each ASCVD outcome are not different.^35^

The effect of lifetime smoking on cardiometabolic risk factors was estimated using random-effects inverse-variance–weighted 2-sample MR (Figure 1). Because the genetic exposure for lifetime smoking index was derived from UK Biobank participants, and MR estimates derived from studies with a high proportion of overlapping samples may be biased, studies of cardiometabolic outcomes that included non–UK Biobank participants were preferred when available.^36^ The genetic exposure for smoking initiation, which included both UK Biobank participants and participants from several other studies, was used in sensitivity analyses to further minimize bias from participant overlap. The MR Steiger test of directionality was performed to validate direction of association between smoking (as the exposure) and cardiometabolic risk factors (as the outcomes).^37^

To determine whether any effect of smoking on ASCVD may be attenuated by effects of smoking on traditional cardiovascular risk factors, multivariable MR was performed. This method can be used to jointly estimate the direct effect of multiple exposure on an outcome, and account for potential violations of MR assumptions (Figure 1).^38,39^ Independent (*r*^2^ = 0.001; distance, 10 000 kilobases) genome-wide significant (*P* < 5 × 10^−8^) variants associated with traditional cardiovascular and inflammatory risk factors (low-density lipoprotein cholesterol level, high-density lipoprotein cholesterol level, level of triglycerides, body mass index, type 2 diabetes, systolic blood pressure, and circulating interleukin 6 levels) were identified using the MR-Base platform. The direct effect of lifetime smoking index was then estimated in models accounting for each traditional risk factor alone and in a model considering all risk factors simultaneously.

### Statistical Analysis

For the primary analysis of smoking on ASCVD outcomes, the statistical significance threshold was set at a 2-sided *P* < .05. The relative associations between lifetime smoking index and the primary ASCVD outcomes were compared using the ratio of the ORs.^35^ For the secondary analysis of smoking on cardiometabolic risk factors, correction for multiple comparisons was made using the Bonferroni method (*P* < .05 / 21 = .002). Cardiometabolic traits with nominal associations (.002 ≤*P* < .05) were considered suggestive. In the multivariable analysis considering the joint effects of smoking and risk factors on ASCVD outcomes, the statistical significance threshold was set at a 2-sided *P* < .05. All analyses were performed using R version 3.6.2 (R Foundation for Statistical Computing). Review of the publications used in the present analysis does not reveal the total recruitment dates for the included cohorts. Data analyses were conducted from August 2019 to June 2020.

## Results

### Selection of Genetic Variants as Proxies for Smoking

Of the 126 independent single-nucleotide variants associated with lifetime smoking index, 116 were available in the summary statistics for stroke, 107 for CAD, and 105 for PAD (eTable 1 in the Supplement). For the lifetime smoking index genetic instrument, the *F* statistic was greater than 10 (range, 25-163; mean, 41), suggesting low risk of weak-instrument bias. To validate the genetic instrument for lifetime smoking index, we performed MR using smoking traits from the GWAS and Sequencing Consortium of Alcohol and Nicotine consortium as outcomes. The lifetime smoking index was significantly associated with increased smoking initiation (OR, 1.66; 95% CI, 1.59-1.73; *P* < .001), smoking cessation (OR, 1.43; 95% CI, 1.37-1.5; *P* < .001), increased amount of smoking (cigarettes per day) (β = 0.514; 95% CI, 0.4-0.63; *P* < .001), and decreased age of smoking initiation (β = −0.38; 95% CI, −0.44 to −0.32) (eFigure 1 in the Supplement).

### Association of Smoking With Risk of ASCVD Outcomes

**Figure 2.**
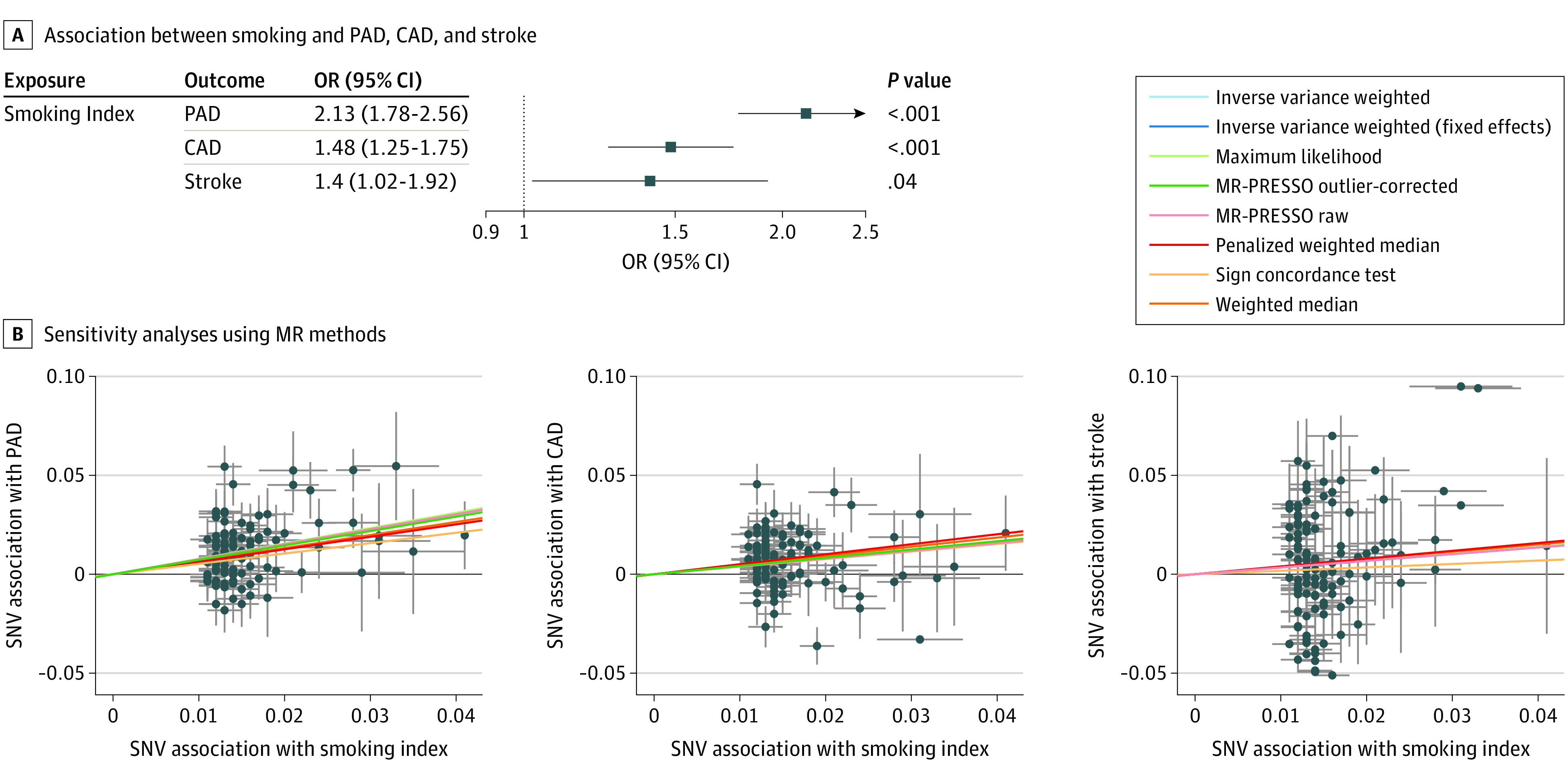
Total Effect Sizes for Associations Between Smoking and Risk of Peripheral Artery Disease (PAD), Coronary Artery Disease (CAD), and Stroke A, In inverse-variance–weighted mendelian randomization, each 1 SD increase in genetic liability to smoking is associated with significantly increased risk of PAD, CAD, and large-artery stroke. Smoking is most strongly associated with increased risk of PAD compared with large-artery stroke (*P* = .04) and CAD (*P* < .001). Odds ratios (ORs) are expressed per 1 SD increase in lifetime smoking index. B, Scatter plots demonstrating the effect of each smoking-associated genetic variant on each atherosclerotic cardiovascular disease outcome on the log-odds scale. Colored lines represent results of each mendelian randomization sensitivity analysis, making different assumptions about horizontal pleiotropy, heterogeneity, and error in the instrument-exposure associations. SNV represents single-nucleotide variant.

In inverse-variance–weighted MR analysis, each 1 SD increase in genetic liability to lifetime smoking index was associated with increased risk of PAD (OR, 2.13; 95% CI, 1.78-2.56; *P* = 3.6 × 10^−16^), CAD (OR, 1.48; 95% CI, 1.25-1.75; *P* = 4.4 × 10^−6^), and stroke (OR, 1.40; 95% CI, 1.02-1.92; *P* = .04) (Figure 2). The Egger bias intercept test identified horizontal pleiotropy for the smoking-CAD pathway (intercept, 0.01; *P* = .046) (eTable 2 in the Supplement) although the findings remained robust in sensitivity analyses using MR methods that account for the possibility of horizontal pleiotropy (Figure 2B; eFigures 2, 3, 4, and 5 in the Supplement), and when using an alternative genetic instrument for smoking (smoking initiation). Genetic liability to smoking initiation was associated with increased risk of PAD (OR, 2.36; 95% CI, 1.81-3.09; *P* < .001), CAD (OR, 1.76; 95% CI, 1.39-2.23; *P* < .001), and stroke (OR, 2.02; 95% CI, 1.33-3.06; *P* < .001) (eFigure 6 in the Supplement).

We next determined whether the estimated effect sizes for the association of smoking differed between the ASCVD endpoints. The primary inverse-variance weighted point estimate for the association of smoking with PAD was significantly greater than the estimates for large-artery stroke (ratio of ORs, 1.52; 95% CI, 1.05-2.19; *P* = .02) or CAD (ratio of ORs, 1.44; 95% CI, 1.12-1.84; *P* = .004).

### Association of Smoking With Risk of Traditional Cardiometabolic Risk Factors

**Figure 3.**
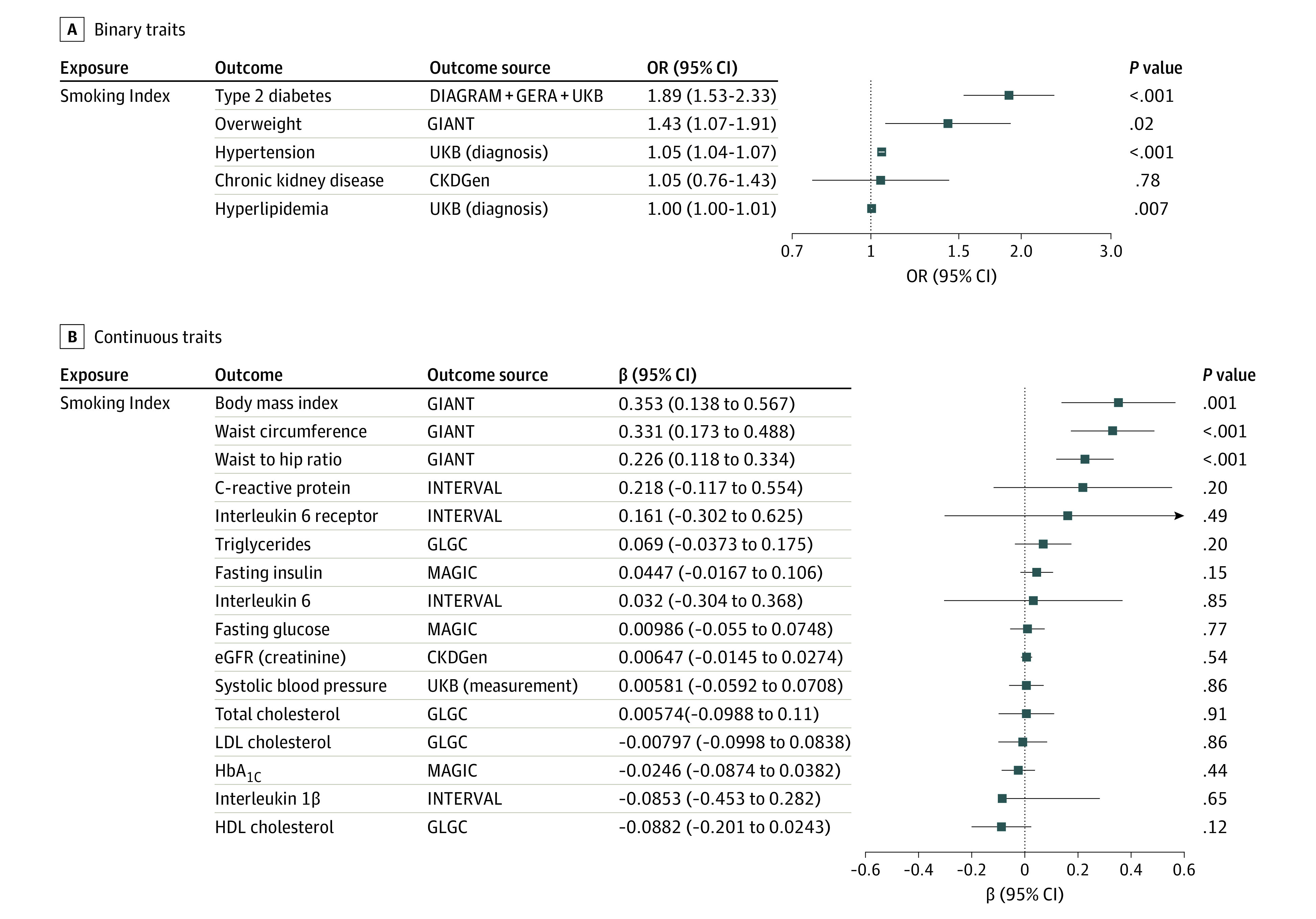
Total Effect Sizes for Associations Between Smoking and Cardiometabolic Risk Factors for Atherosclerotic Cardiovascular Disease Inverse-variance–weighted mendelian randomization was performed to determine whether genetic liability to smoking altered risk of cardiometabolic risk factors for atherosclerotic cardiovascular disease. Genetic liability to smoking increased risk of both (A) binary traits and (B) continuous traits that are common risk factors for cardiometabolic disease. Effect estimates are expressed per 1 SD increase in lifetime smoking index. CKDGen indicates Chronic Kidney Disease Genetics Consortium; DIAGRAM, Diabetes Genetics Replication and Meta-analysis consortium; eGFR, estimated glomerular filtration rate; GERA, Genetic Epidemiology Research on Aging; GIANT, Genetic Investigation of Anthropometric Traits Consortium; GLGC, Global Lipid Genetics Consortium; HbA_1C_, hemoglobin A_1C_; HDL, high-density lipoprotein; LDL, low-density lipoprotein; MAGIC, Meta-Analyses of Glucose and Insulin-Related Traits Consortium; OR, odds ratio; and UKB, UK Biobank.

We next considered the effect of increasing genetic liability to lifetime smoking on other cardiometabolic traits that are known ASCVD risk factors. Increasing genetic liability to lifetime smoking was significantly associated (*P* < .001) with increased risk of type 2 diabetes (OR, 1.89; 95% CI, 1.53-2.33), hypertension (OR, 1.05; 95% CI, 1.04-1.07), waist circumference (β = 0.33; 95% CI, 0.17-0.49), body mass index (β = 0.35; 95% CI, 0.14-0.57), and waist to hip ratio (β = 0.23; 95% CI, 0.12-0.33), with a suggestive (*P* < .05) increase in the risk of hyperlipidemia (OR, 1.00; 95% CI, 1.00-1.01) and risk of being overweight (OR, 1.43; 95% CI, 1.07-1.91) (Figure 3). In a sensitivity analysis considering an alternative genetic instrument for smoking, the results were similar (eFigure 7 in the Supplement). The MR Steiger test confirmed the directionality of these significant and suggestive findings (*P* < .001) (eTable 3 in the Supplement).

### Influence of Traditional Cardiometabolic Risk Factors on Smoking-ASCVD Relationship

**Figure 4.**
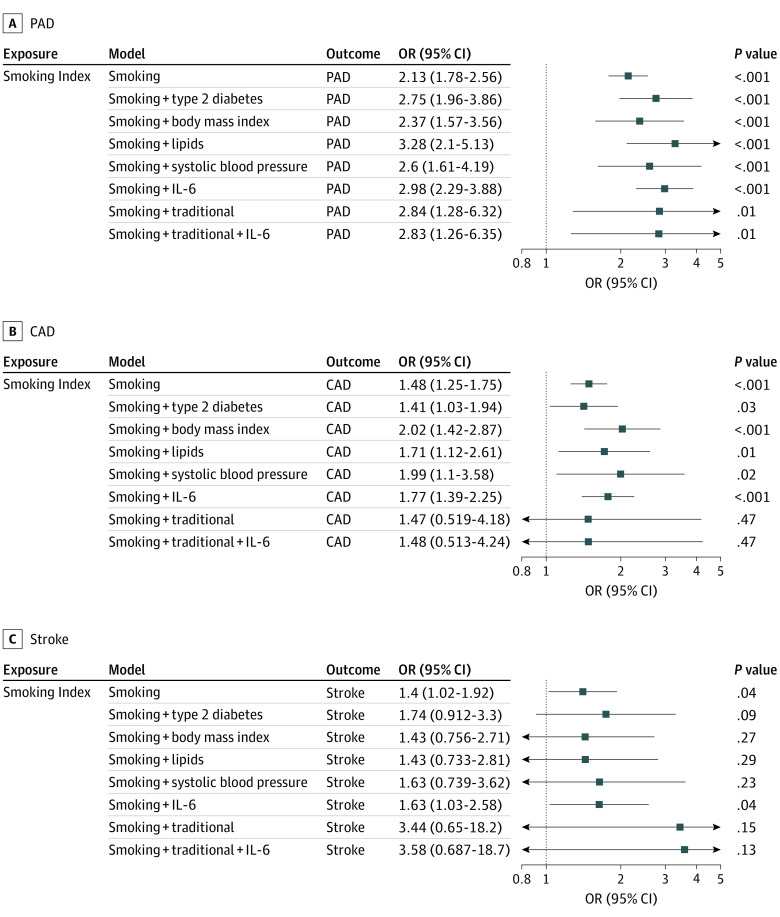
Direct Effect Sizes for Associations Between Smoking and Risk of Peripheral Artery Disease (PAD), Coronary Artery Disease (CAD), and Stroke Multivariable mendelian randomization was performed to estimate the direct effect of the association of smoking with atherosclerotic cardiovascular disease after accounting for the effects of smoking on other cardiovascular risk factors. The estimated direct effects of smoking on PAD, CAD, and stroke are not substantially attenuated in models adjusting for traditional cardiovascular risk factors (type 2 diabetes, body mass index, lipids [high-density lipoprotein cholesterol, low-density lipoprotein cholesterol, and triglycerides], and systolic blood pressure), or inflammation (interleukin 6 [IL-6] levels). Odds ratios (ORs) are expressed per 1 SD increase in lifetime smoking index.

To evaluate whether increasing genetic liability to smoking was directly associated with increased risk of ASCVD, or whether the association was attenuated after accounting for traditional cardiovascular risk factors (type 2 diabetes, lipids, body mass index, and systolic blood pressure), we performed multivariable MR. Increasing genetic liability to smoking was associated with increased risk of PAD, CAD, and stroke, after accounting for effects of smoking on each risk factor independently, and in a combined model considering all risk factors (Figure 4). The point estimates for the association of smoking with ASCVD were not substantially attenuated after accounting for traditional risk factors (PAD: OR, 2.84 [95% CI, 1.28-6.32]; *P* = .01; CAD: OR, 1.47 [95% CI, 0.52-4.18]; *P* = .47; stroke: OR, 3.44 [95% CI, 0.65-18.20]; *P* = .15), or in a model further including interleukin 6 as a marker of inflammatory risk of ASCVD (PAD: OR, 2.83 [95% CI, 1.26-6.35]; *P* = .01; CAD: OR, 1.48 [95% CI, 0.51-4.24]; *P* = .47; stroke: OR, 3.58 [95% CI, 0.69-18.70]; *P* = .13) compared with the univariate estimates.

## Discussion

Using MR, we leveraged population-scale human genetics to estimate a potentially causal association between smoking, cardiometabolic risk factors, and ASCVD outcomes across diverse vascular beds (CAD, PAD, and stroke). By drawing from several large GWASs, we were able to consider more cases by an order of magnitude for smoking, cardiometabolic risk factors, and ASCVD outcomes compared with previous observational studies. Although observational studies remain at risk of bias due to residual confounding, the genetic instrumental variables used here in the MR framework provided effect estimates that were less susceptible to reverse causality and confounding from environmental factors. Because genetic variants are randomly assorted during meiosis, mimicking randomization in a clinical trial, we were able to estimate potentially causal relationships between smoking and cardiometabolic traits.

Our results suggested that smoking had a direct atherogenic effect that varied across vascular beds. This finding is largely consistent with prior investigations of smoking on ASCVD. Although the association between smoking and ASCVD has been shown previously in observational studies, our MR analysis provides strong evidence that may be consistent with a causal relationship. Our finding that smoking appears to more strongly influence the risk of PAD compared with CAD or stroke is consistent with recent results from the ARIC (Atherosclerosis Risk in Communities) study cohort, in which the effect of smoking was greatest for PAD, and a recent MR analysis of UK Biobank participants demonstrating a strong effect of smoking on PAD.^4,40^ Although the mechanism behind the stronger relationship between smoking and PAD is not clear, structural and functional differences within the vascular beds and the complex interplay between smoking and other ASCVD risk factors may contribute.^41,42^ For example, although both acute PAD and CAD events typically result from luminal thrombosis, and both diseases have atherosclerotic manifestations, typical acute CAD lesions occur in the setting of atherothrombosis, while acute PAD-associated lesions more typically result from in situ thrombosis or embolism.^43^ Indeed, a strong association between the factor V Leiden variant (F5 p.R506Q) and PAD has been identified; however, this association is not present for CAD, raising the possibility that smoking-related changes in coagulation may explain some of the differential associations between smoking and ASCVD outcomes.^9,44^ Further understanding of the mechanistic differences in ASCVD pathophysiology across vascular beds may ultimately lead to more targeted prevention and treatment strategies.

Genetic liability to smoking is also associated with cardiometabolic traits that are themselves risk factors ASCVD. The MR finding that increasing genetic liability to smoking is associated with type 2 diabetes is consistent with recent observational and MR studies.^6,45,46,47,48^ We also identified increasing genetic liability to smoking as a risk factor for hypertension and increased waist circumference, body mass index, and waist to hip ratio, although prior studies have identified conflicting effects of smoking on these traits.^49,50,51,52,53,54^ A prior single-sample MR analysis from the Nord-Trøndelag Health Study (HUNT Study) found a protective effect of smoking on body mass index, waist circumference, and hip circumference but found no associations with blood pressure, levels of lipids, or glucose levels.^51^ Their study may have been limited by the single-sample design, modest study size, and weak single single-nucleotide variant (rs1051730) instrument for smoking, which all may have contributed to bias toward observational estimates.^12^ More recent MR studies have corroborated our finding that smoking traits are associated with increased body mass index.^48^ Conflict among observational studies may be related to residual confounding or reverse causality. Mendelian randomization assumes that genetic variants proxying an exposure produce similar effects to the exposure itself, although this assumption may not always be valid. For example, lifetime exposure to adverse genetics may have different health consequences when compared with more concentrated environmental exposures, highlighted by the much larger protective effects of genetically lower low-density lipoprotein cholesterol level and systolic blood pressure on risk of coronary heart disease in comparison with effect estimates from randomized trials of treatments for these risk factors.^55,56^

The effect of increasing genetic liability to smoking on ASCVD outcomes appears to be independent from the effects of smoking on traditional cardiovascular risk factors. The point estimate of the direct effect of smoking (when jointly considering smoking and cardiometabolic risk factors) was similar (or greater) than the total effect, suggesting the possibility of causal interaction between smoking and traditional risk factors, which could be investigated using factorial MR in a single-sample setting.^57^ Proposed mechanisms by which smoking may independently contribute to cardiovascular events include among others hypercoagulability, increased myocardial work, decreased oxygen delivery (due to elevated carboxyhemoglobin levels), coronary vasoconstriction, and increased catecholamine levels.^57^

The finding that smoking confers strong independent risk for ASCVD even when considering other traditional cardiovascular risk factors has important public health implications. More precise estimation of the effect of smoking on ASCVD outcomes may help calibrate the expected benefit of smoking cessation initiatives, and efforts to reduce the burden of cardiovascular disease should continue to focus on smoking cessation. Further, public awareness of ASCVD varies across outcomes and is particularly low for PAD.^58^ The current analysis provides strong genetic evidence for the effects of smoking and other ASCVD risk factors on CAD, PAD, and stroke and may be useful in raising public understanding of these risk factor–outcome relationships.

### Limitations

The current study must be interpreted within the context of its limitations. The study focused primarily on individuals of European ancestry, which may limit generalization to other populations, highlighting the need for genomic studies in diverse ancestral groups. The MR framework relies on a key assumption that the risk conferred by an exposure is equivalent whether mediated by genetics or environment, and that genetic risk is conferred through the exposure of interest rather than via pleiotropic effects.^12^ Although findings were consistent in sensitivity analyses using MR methods robust to the presence of pleiotropy, there may be gene-environment interactions, such as those previously shown at the *ADAMTS7* locus for CAD and at the *CHRNA3* locus for PAD, that modify and alter the relationship between smoking and ASCVD outcomes.^9,59^ Although differences in the underlying structure of the ASCVD studies could affect the estimate of differential risk between the ASCVD outcomes, the 2-sample MR framework tends to bias causal estimates toward the null, lending further confidence in our overall finding that smoking was strongly associated with increased risk of all ASCVD outcomes. Similarly, differences in the ascertainment of ASCVD and cardiometabolic traits (eg, inclusion of incident vs prevalent disease) may lead to biased estimates owing to prevalence-incidence or to selective-survival bias although the analyzed cohorts in the present study included predominately prevalent disease, which would be expected to bias estimates toward the null. Finally, future study of additional smoking-related traits, such as duration or quantity of smoking and smoking cessation, and other MR methods may provide additional insight into potential differential effects of these traits in different vascular beds, clarifying recent observational findings that these traits may affect ASCVD risk.^4,60^

## Conclusions

In this MR study using genetic data from large studies of smoking, atherosclerosis, and cardiometabolic disease, genetic liability to smoking was associated with increased risk of ASCVD, with the strongest association between smoking and PAD, independent from traditional cardiovascular risk factors.

## Notes

Supplement. **eFigure 1.** MR of Lifetime Smoking Index to GSCAN Smoking Traits **eFigure 2.** Primary Smoking-ASCVD MR Funnel Plots **eFigure 3.** Primary Smoking-ASCVD MR Leave-One-Out Analysis **eFigure 4.** Primary Smoking-ASCVD MR Single-SNP Analysis **eFigure 5.** Primary Smoking-ASCVD MR Single-SNP Analysis **eFigure 6.** MR of Smoking Initiation to ASCVD Outcomes **eFigure 7.** MR of Smoking Initiation to Cardiometabolic Risk Factors **eTable 1.** Smoking Index exposure SNPs and Corresponding SNP Effects for CAD, PAD, and Stroke Outcomes **eTable 2.** Egger Bias Intercept Test for Smoking Index-ASCVD Analysis **eTable 3.** MR Steiger Directionality Test for Smoking-Cardiometabolic Risk Factor Analysis
