# Supplementary material for: Genetics of Smoking and Risk of Atherosclerotic Cardiovascular Diseases: A Mendelian Randomization Study
Source: JAMA Netw Open. 2021 Jan 19;4(1):e2034461. doi: 10.1001/jamanetworkopen.2020.34461 (PMC7816104; doi:10.1001/jamanetworkopen.2020.34461)
Supplement: Supplement. — eFigure 1. MR of Lifetime Smoking Index to GSCAN Smoking Traits eFigure 2. Primary Smoking-ASCVD MR Funnel Plots eFigure 3. Primary Smoking-ASCVD MR Leave-One-Out Analysis eFigure 4. Primary Smoking-ASCVD MR Single-SNP Analysis eFigure 5. Primary Smoking-ASCVD MR Single-SNP Analysis eFigure 6. MR of Smoking Initiation to ASCVD Outcomes eFigure 7. MR of Smoking Initiation to Cardiometabolic Risk Factors eTable 1. Smoking Index exposure SNPs and Corresponding SNP Effects for CAD, PAD, and Stroke Outcomes eTable 2. Egger Bias Intercept Test for Smoking Index-ASCVD Analysis eTable 3. MR Steiger Directionality Test for Smoking-Cardiometabolic Risk Factor Analysis [file jamanetwopen-e2034461-s001.pdf]

Supplementary Online Content

Levin MG, Klarin D, Assimes TL, et al; VA Million Veteran Program. Genetics of smoking and risk of atherosclerotic cardiovascular diseases: a mendelian randomization study. *JAMA Netw Open*. 2021;4(1):e2034461. doi:10.1001/jamanetworkopen.2020.34461

eFigure 1. MR of Lifetime Smoking Index to GSCAN Smoking Traits
eFigure 2. Primary Smoking-ASCVD MR Funnel Plots
eFigure 3. Primary Smoking-ASCVD MR Leave-One-Out Analysis
eFigure 4. Primary Smoking-ASCVD MR Single-SNP Analysis
eFigure 5. Primary Smoking-ASCVD MR Single-SNP Analysis
eFigure 6. MR of Smoking Initiation to ASCVD Outcomes
eFigure 7. MR of Smoking Initiation to Cardiometabolic Risk Factors
eTable 1. Smoking Index exposure SNPs and Corresponding SNP Effects for CAD, PAD, and Stroke Outcomes
eTable 2. Egger Bias Intercept Test for Smoking Index-ASCVD Analysis
eTable 3. MR Steiger Directionality Test for Smoking-Cardiometabolic Risk Factor Analysis

This supplementary material has been provided by the authors to give readers additional information about their work.

© 2021 Levin MG et al. *JAMA Network Open*.

eFigure 1. MR of Lifetime Smoking Index to GSCAN Smoking Traits

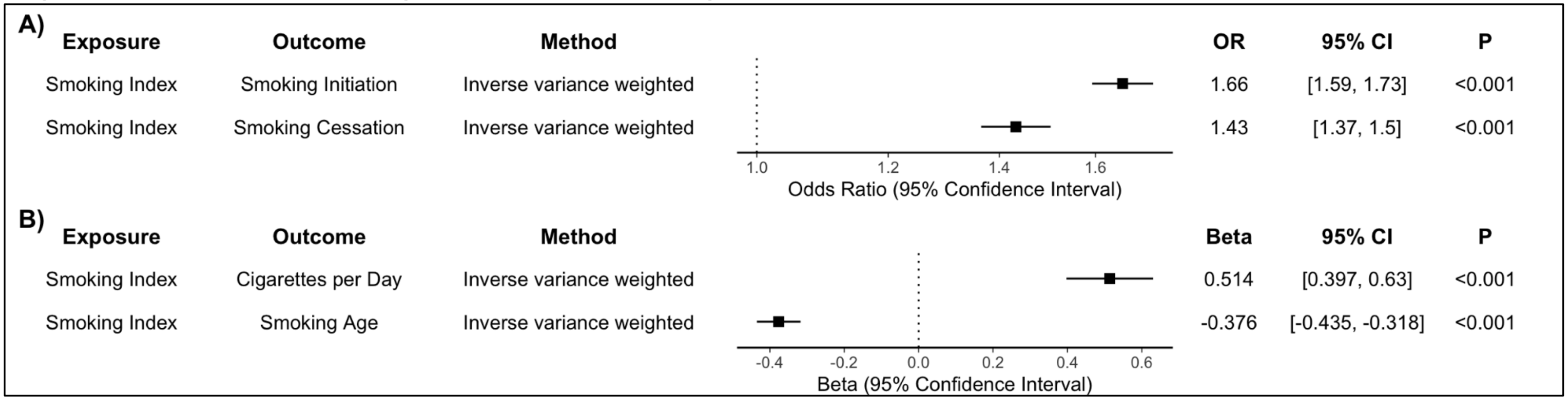

The lifetime smoking instrument was validated against smoking exposures from the GSCAN consortium. A) Odds ratios for the effect of each one standard deviation increase in lifetime smoking on Smoking Initiation and Smoking Cessation. B) Beta coefficients for the effect of each one standard deviation increase in lifetime smoking on Cigarettes per Day and Smoking Age (age of initiation). OR = Odds Ratio, CI = Confidence Interval.

© 2021 Levin MG et al. *JAMA Network Open*.

eFigure 2. Primary Smoking-ASCVD MR Funnel Plots

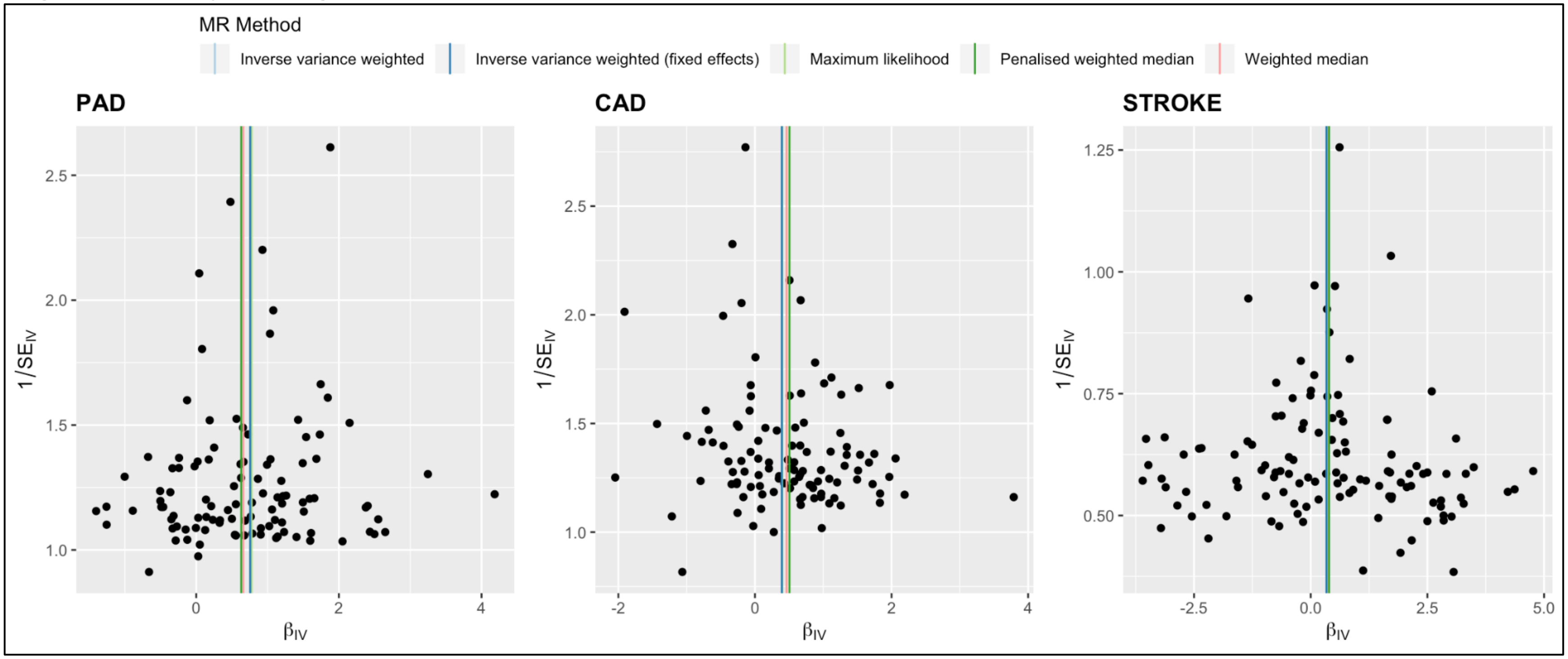

Funnel plots showing the relationship between the SNP-level causal effects of lifetime smoking index on each ASCVD outcome (PAD, CAD, and large artery stroke) against the inverse of the standard error of the causal estimate. Vertical lines represent the MR estimates using all SNPs for each of five different MR methods. Asymmetry in funnel plots may be an indicator of heterogeneous effects that may be related to directional horizontal pleiotropy or "winner's curse" that lead to bias in effect estimates.

© 2021 Levin MG et al. *JAMA Network Open*.

eFigure 3. Primary Smoking-ASCVD MR Leave-One-Out Analysis

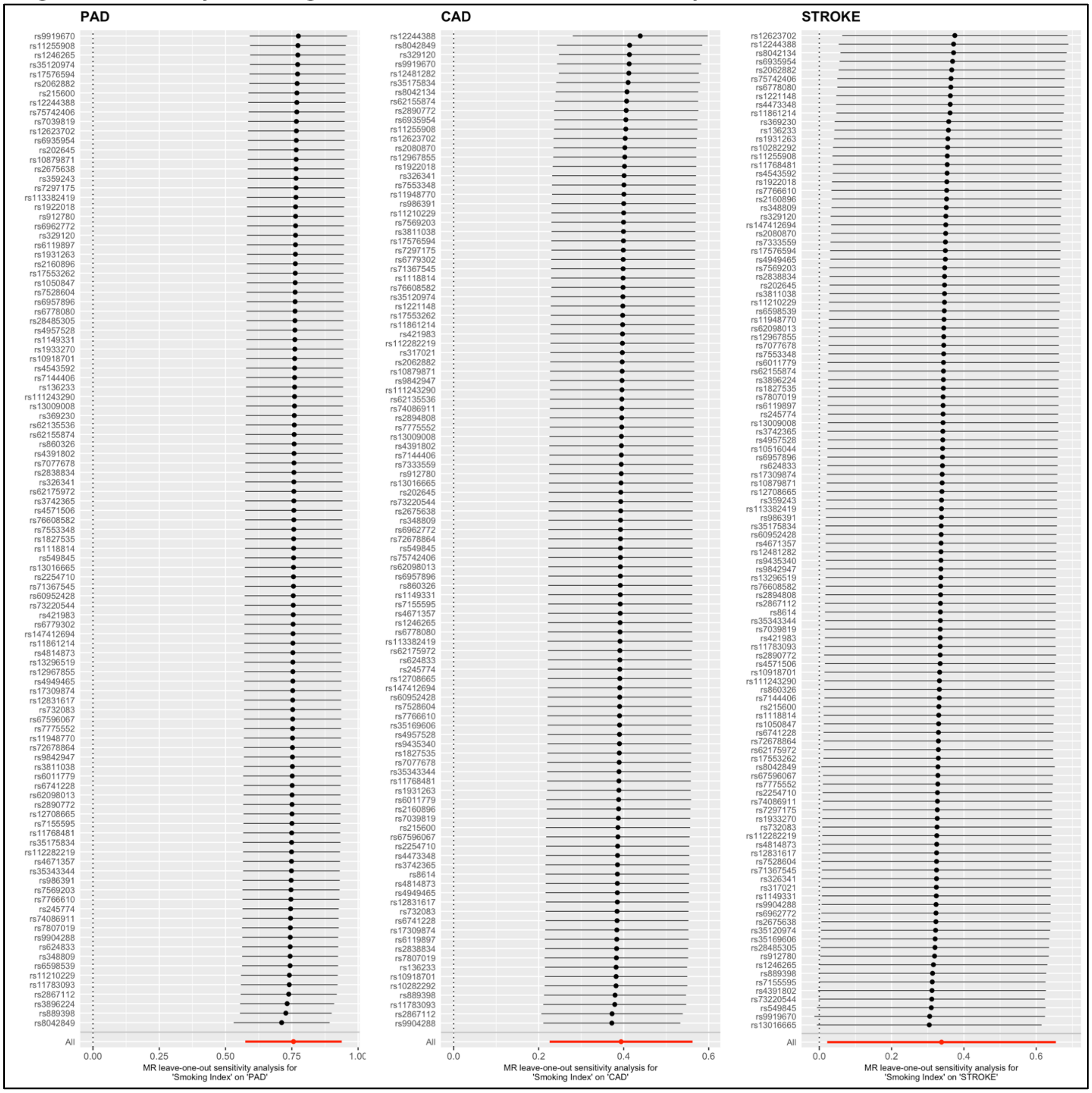

In leave-one-out analysis, each individual SNP is sequentially excluded from the inverse variance weighted MR model to identify outlier SNPs that may drive outsize effects. Plotted are the log odds ratios of each leave-one-out model for each ASCVD outcome (CAD, PAD, and large artery stroke) against the SNP excluded from each model. Lines extending from each point represent the 95% confidence intervals of the effect estimate.

© 2021 Levin MG et al. *JAMA Network Open*.

eFigure 4. Primary Smoking-ASCVD MR Single-SNP Analysis

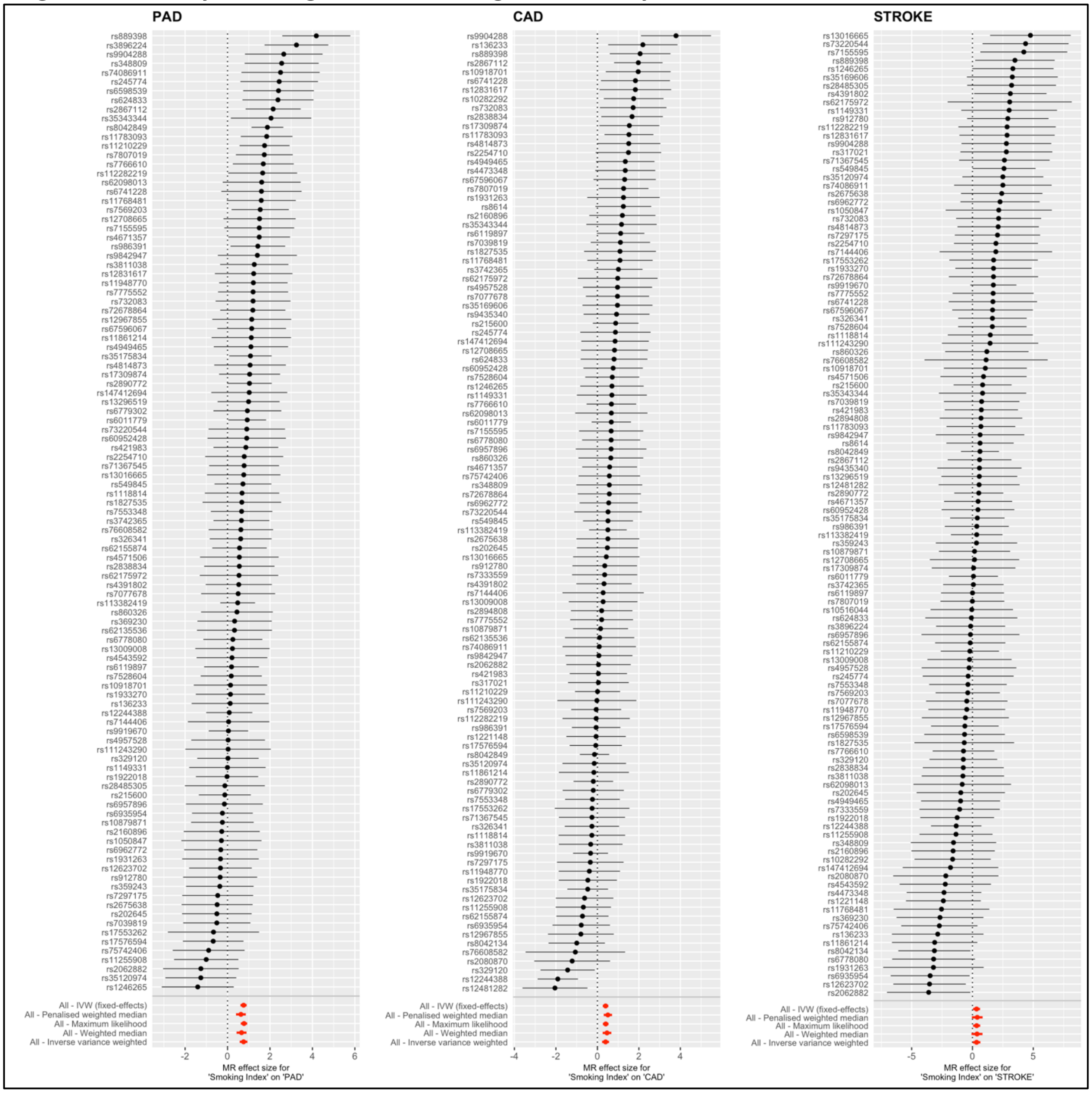

The single-SNP MR method plots the Wald estimate of the effect of each SNP included in the lifetime smoking genetic instrument on each ASCVD outcome. Lines extending from each point represent the 95% confidence interval of the odds ratio (points). Red estimates at the bottom of each plot represent the summary measures from five MR models including all of the individual SNPs.

© 2021 Levin MG et al. *JAMA Network Open*.

eFigure 5. Primary Smoking-ASCVD MR Single-SNP Analysis

Exposure	Outcome	MR Method	OR	95% CI	P
Smoking Index	CAD	Weighted median	1.59	[1.3-1.94]	4×10^{-6}
Smoking Index	CAD	Penalised weighted median	1.66	[1.33-2.06]	5×10^{-6}
Smoking Index	CAD	MR-PRESSO Raw	1.48	[1.25-1.75]	1×10^{-5}
Smoking Index	CAD	MR-PRESSO Outlier-corrected	1.52	[1.3-1.77]	4×10^{-7}
Smoking Index	CAD	Maximum likelihood	1.49	[1.3-1.71]	1×10^{-8}
Smoking Index	CAD	IVW (fixed-effects)	1.48	[1.3-1.7]	1×10^{-8}
Smoking Index	PAD	Weighted median	1.94	[1.53-2.44]	3×10^{-8}
Smoking Index	PAD	Penalised weighted median	1.88	[1.48-2.39]	3×10^{-7}
Smoking Index	PAD	MR-PRESSO Raw	2.13	[1.78-2.56]	9×10^{-13}
Smoking Index	PAD	MR-PRESSO Outlier-corrected	2.07	[1.74-2.46]	6×10^{-13}
Smoking Index	PAD	Maximum likelihood	2.17	[1.87-2.53]	5×10^{-24}
Smoking Index	PAD	IVW (fixed-effects)	2.13	[1.84-2.47]	6×10^{-24}
Smoking Index	STROKE	Weighted median	1.46	[0.941-2.25]	0.09
Smoking Index	STROKE	Penalised weighted median	1.48	[0.938-2.34]	0.09
Smoking Index	STROKE	MR-PRESSO Raw	1.4	[1.02-1.92]	0.04
Smoking Index	STROKE	Maximum likelihood	1.41	[1.05-1.89]	0.02
Smoking Index	STROKE	IVW (fixed-effects)	1.4	[1.05-1.87]	0.02

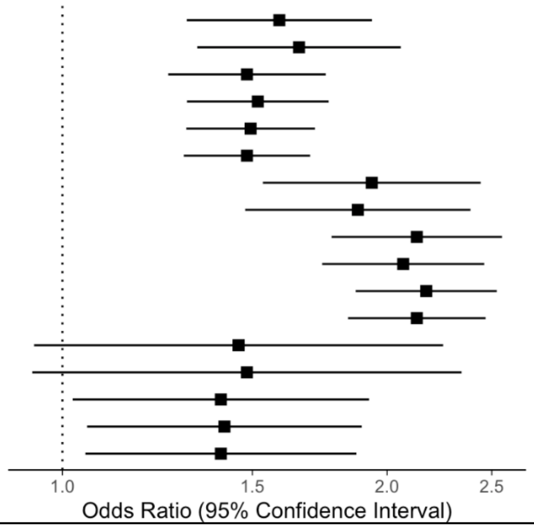

The effect of each one standard deviation increase in lifetime smoking index on each ASCVD outcome (CAD, PAD, and large artery stroke) was estimated using multiple MR methods that make different assumptions about the potential violations of the instrumental variable assumptions, heterogeneity, and error in the instrument-exposure associations. The effect of smoking on each outcome was consistent across MR models.

© 2021 Levin MG et al. *JAMA Network Open*.

eFigure 6. MR of Smoking Initiation to ASCVD Outcomes

Exposure	Outcome	Method	OR	95% CI	P
Smoking Initiation	PAD	Inverse variance weighted	2.36	[1.81-3.09]	4×10^{-10}
Smoking Initiation	PAD	IVW (fixed-effects)	2.36	[1.89-2.95]	4×10^{-14}
Smoking Initiation	PAD	Maximum likelihood	2.45	[1.95-3.09]	2×10^{-14}
Smoking Initiation	PAD	Penalised weighted median	2.06	[1.45-2.95]	6×10^{-5}
Smoking Initiation	PAD	Weighted median	2.08	[1.44-3]	1×10^{-4}
Smoking Initiation	STROKE	Inverse variance weighted	2.02	[1.33-3.06]	9×10^{-4}
Smoking Initiation	STROKE	IVW (fixed-effects)	2.02	[1.33-3.06]	9×10^{-4}
Smoking Initiation	STROKE	Maximum likelihood	2.08	[1.36-3.17]	7×10^{-4}
Smoking Initiation	STROKE	Penalised weighted median	1.37	[0.742-2.51]	0.3
Smoking Initiation	STROKE	Weighted median	1.44	[0.799-2.59]	0.2
Smoking Initiation	CAD	Inverse variance weighted	1.76	[1.39-2.23]	3×10^{-6}
Smoking Initiation	CAD	IVW (fixed-effects)	1.76	[1.43-2.16]	1×10^{-7}
Smoking Initiation	CAD	Maximum likelihood	1.8	[1.45-2.22]	7×10^{-8}
Smoking Initiation	CAD	Penalised weighted median	1.79	[1.3-2.46]	4×10^{-4}
Smoking Initiation	CAD	Weighted median	1.79	[1.3-2.47]	3×10^{-4}

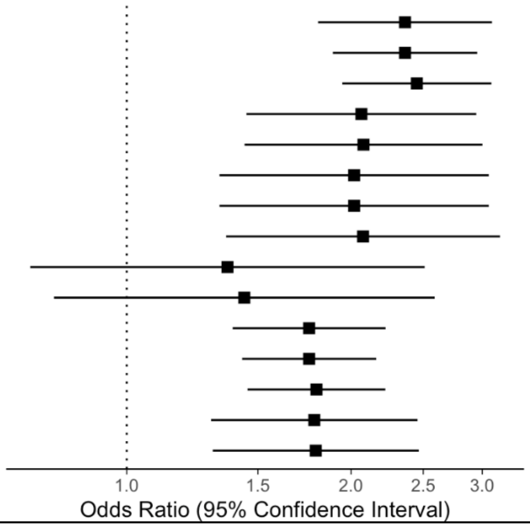

The robustness of the effect of lifetime smoking on ASCVD outcomes was assessed using an additional genetic instrument for smoking (Smoking Initiation). The effect of each one standard deviation increase in smoking initiation on each ASCVD outcome (CAD, PAD, and large artery stroke) was estimated using multiple MR methods that make different assumptions about the potential violations of the instrumental variable assumptions, heterogeneity, and error in the instrument-exposure associations.

© 2021 Levin MG et al. *JAMA Network Open*.

eFigure 7. MR of Smoking Initiation to Cardiometabolic Risk Factors

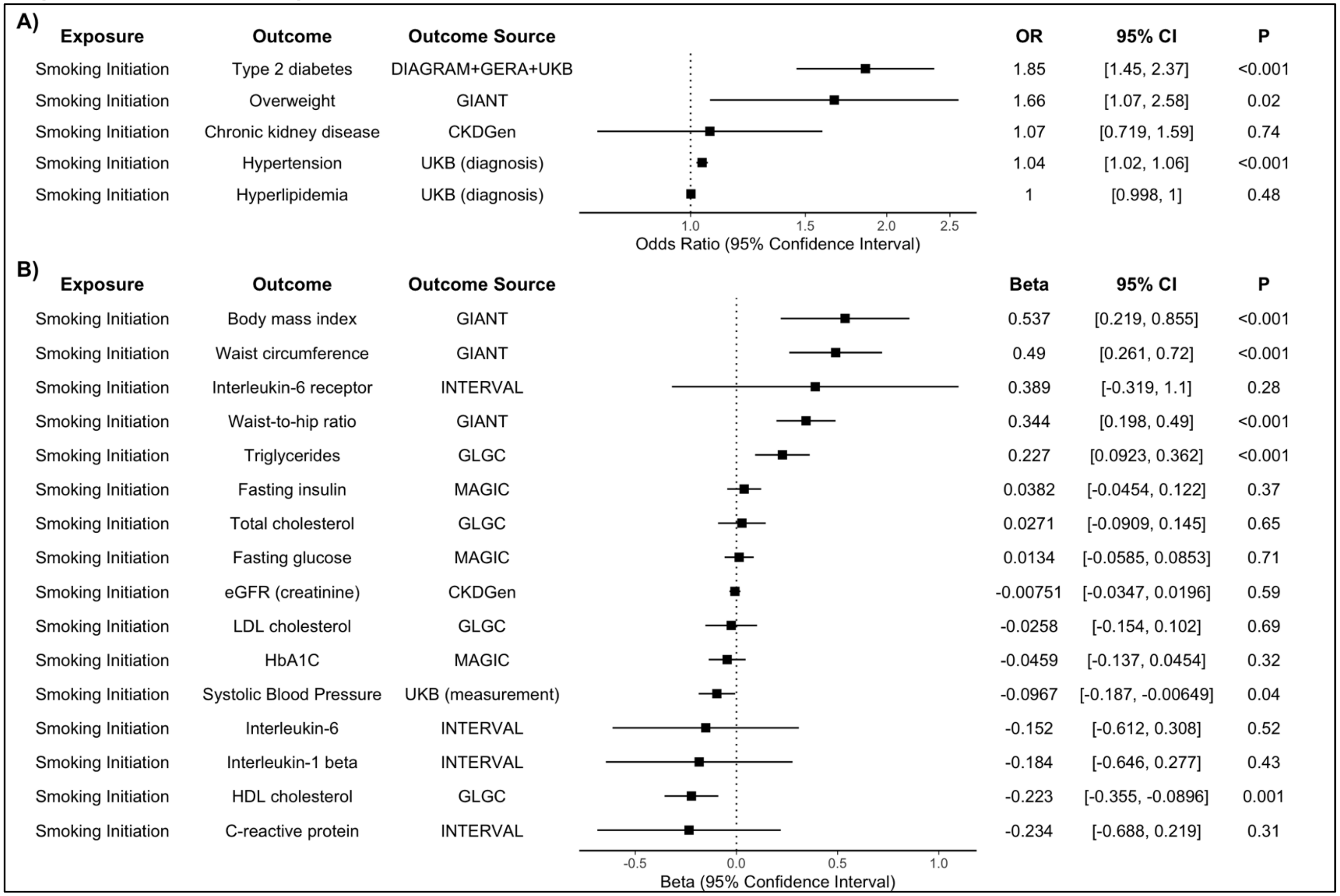

The robustness of the effect of lifetime smoking on cardiometabolic outcomes was assessed using an additional genetic instrument for smoking (Smoking Initiation). The effect of each one standard deviation increase in smoking initiation on each cardiometabolic outcome (CAD, PAD, and large artery stroke) was estimated using inverse variance weighted MR. Genetic liability to smoking increased risk of both **(A)** binary traits, and **(B)** continuous traits that are common risk factors for cardiometabolic disease. OR = Odds Ratio, CI = Confidence Interval.

© 2021 Levin MG et al. *JAMA Network Open*.

eTable 1. Smoking Index exposure SNPs and Corresponding SNP Effects for CAD, PAD, and Stroke Outcomes

Exposure	Outcome	SNP	Effect allele	Alternate allele	EAF exposure	Beta exposure	SE exposure	P exposure	Beta outcome	SE outcome	P outcome
Smoking Index	PAD	rs1050847	C	T	0.426	0.011	0.002	1.80E-08	-0.003166	0.0106	0.7651
Smoking Index	PAD	rs10879871	T	G	0.343	-0.014	0.002	5.60E-11	0.003389	0.01054	0.7479
Smoking Index	PAD	rs10918701	G	A	0.372	0.012	0.002	2.00E-08	0.00169	0.0106	0.8734
Smoking Index	PAD	rs111243290	T	C	0.963	-0.029	0.005	4.30E-08	-0.00081	0.02976	0.9783
Smoking Index	PAD	rs1118814	G	A	0.324	0.012	0.002	1.40E-08	0.008215	0.01074	0.4446
Smoking Index	PAD	rs11210229	A	G	0.384	0.017	0.002	2.80E-16	0.02966	0.01022	0.003727
Smoking Index	PAD	rs112282219	G	A	0.959	-0.033	0.005	3.70E-11	-0.05464	0.02734	0.04676
Smoking Index	PAD	rs11255908	T	G	0.743	-0.015	0.002	1.90E-10	0.01505	0.0116	0.194
Smoking Index	PAD	rs113382419	C	A	0.889	-0.041	0.003	9.70E-38	-0.01966	0.01713	0.2519
Smoking Index	PAD	rs1149331	G	A	0.477	-0.011	0.002	3.20E-08	0.00005096	0.01011	0.996
Smoking Index	PAD	rs11768481	C	A	0.666	0.013	0.002	1.00E-09	0.02067	0.01079	0.05516
Smoking Index	PAD	rs11783093	C	T	0.839	0.023	0.003	1.00E-16	0.04243	0.01429	0.002903
Smoking Index	PAD	rs11861214	G	T	0.784	0.013	0.002	2.60E-08	0.01457	0.01241	0.24
Smoking Index	PAD	rs11948770	T	C	0.768	-0.015	0.002	4.00E-10	-0.01825	0.01234	0.1393
Smoking Index	PAD	rs12244388	G	A	0.661	-0.019	0.002	9.60E-20	-0.001552	0.01053	0.8829
Smoking Index	PAD	rs1246265	T	C	0.305	-0.013	0.002	5.30E-09	0.01824	0.01125	0.1051
Smoking Index	PAD	rs12623702	A	G	0.613	-0.014	0.002	5.70E-12	0.004671	0.01055	0.6578
Smoking Index	PAD	rs12708665	A	G	0.285	-0.013	0.002	3.40E-09	-0.01961	0.01127	0.08163

© 2021 Levin MG et al. *JAMA Network Open*.

Smoking Index	PAD	rs12831617	C	T	0.764	-0.013	0.002	2.40E-08	-0.01601	0.01213	0.1872
Smoking Index	PAD	rs12967855	A	G	0.331	0.012	0.002	4.00E-08	0.0137	0.01139	0.2295
Smoking Index	PAD	rs13009008	A	G	0.328	0.012	0.002	6.40E-09	0.002786	0.01071	0.7948
Smoking Index	PAD	rs13016665	C	A	0.577	-0.012	0.002	1.40E-09	-0.009174	0.01059	0.3866
Smoking Index	PAD	rs13296519	G	T	0.606	-0.014	0.002	1.40E-11	-0.0139	0.01044	0.1831
Smoking Index	PAD	rs136233	A	G	0.809	-0.014	0.003	2.70E-08	-0.001754	0.01297	0.8924
Smoking Index	PAD	rs147412694	G	A	0.85	-0.017	0.003	2.30E-09	-0.0174	0.01552	0.2629
Smoking Index	PAD	rs17309874	G	A	0.74	-0.016	0.002	5.60E-13	-0.01666	0.01174	0.1561
Smoking Index	PAD	rs17553262	A	C	0.885	-0.018	0.003	6.90E-09	0.01191	0.01974	0.546
Smoking Index	PAD	rs17576594	G	A	0.724	0.016	0.002	1.90E-12	-0.0108	0.01166	0.3548
Smoking Index	PAD	rs1827535	T	G	0.686	-0.012	0.002	1.20E-08	-0.008078	0.01134	0.4764
Smoking Index	PAD	rs1922018	C	T	0.364	0.014	0.002	2.50E-12	-0.0003203	0.01049	0.9757
Smoking Index	PAD	rs1931263	G	T	0.51	-0.011	0.002	4.00E-08	0.003646	0.01013	0.7189
Smoking Index	PAD	rs1933270	T	G	0.364	0.013	0.002	1.70E-10	0.00178	0.01082	0.8694
Smoking Index	PAD	rs202645	A	G	0.203	-0.015	0.002	5.00E-09	0.007542	0.01254	0.5478
Smoking Index	PAD	rs2062882	G	A	0.587	-0.012	0.002	1.00E-08	0.01507	0.0109	0.1668
Smoking Index	PAD	rs215600	G	A	0.358	0.017	0.002	2.00E-15	-0.002176	0.01063	0.8378
Smoking Index	PAD	rs2160896	C	T	0.317	-0.012	0.002	2.20E-08	0.003263	0.01097	0.7662
Smoking Index	PAD	rs2254710	C	A	0.236	0.013	0.002	3.00E-08	0.01022	0.0122	0.4026
Smoking Index	PAD	rs245774	A	G	0.272	-0.013	0.002	9.00E-09	-0.03165	0.01212	0.008915
Smoking Index	PAD	rs2675638	G	A	0.581	0.012	0.002	1.20E-09	-0.005877	0.01024	0.5661
Smoking Index	PAD	rs2838834	C	T	0.699	-0.013	0.002	9.20E-10	-0.00723	0.01099	0.5108
Smoking Index	PAD	rs28485305	C	T	0.631	0.011	0.002	2.80E-08	-0.001375	0.01057	0.8965
Smoking Index	PAD	rs2867112	T	G	0.835	0.021	0.003	8.30E-15	0.04515	0.01392	0.001137
Smoking Index	PAD	rs2890772	G	T	0.413	-0.02	0.002	3.60E-22	-0.02068	0.01072	0.05367

© 2021 Levin MG et al. *JAMA Network Open*.

Smoking Index	PAD	rs326341	G	A	0.525	0.014	0.002	8.70E-12	0.008683	0.01042	0.4048
Smoking Index	PAD	rs329120	C	T	0.581	0.014	0.002	5.80E-12	0.0002788	0.01034	0.9785
Smoking Index	PAD	rs348809	A	G	0.348	-0.012	0.002	1.90E-08	-0.03062	0.01069	0.004137
Smoking Index	PAD	rs35120974	A	G	0.583	-0.012	0.002	2.10E-09	0.0151	0.01023	0.1397
Smoking Index	PAD	rs35175834	G	A	0.788	-0.024	0.002	4.80E-22	-0.02592	0.01225	0.0346
Smoking Index	PAD	rs35343344	C	A	0.733	0.013	0.002	1.00E-08	0.02666	0.01257	0.03366
Smoking Index	PAD	rs359243	T	C	0.393	-0.013	0.002	8.30E-10	0.00472	0.01056	0.655
Smoking Index	PAD	rs369230	G	T	0.308	-0.013	0.002	2.00E-09	-0.004333	0.01162	0.7092
Smoking Index	PAD	rs3742365	T	C	0.595	-0.016	0.002	2.30E-14	-0.01048	0.01074	0.3294
Smoking Index	PAD	rs3811038	T	C	0.724	-0.014	0.002	9.20E-10	-0.01765	0.0115	0.1252
Smoking Index	PAD	rs3896224	A	G	0.585	0.014	0.002	1.00E-11	0.04547	0.01074	0.00002261
Smoking Index	PAD	rs421983	T	C	0.519	0.013	0.002	3.10E-10	0.01124	0.01012	0.2664
Smoking Index	PAD	rs4391802	A	G	0.707	0.015	0.002	1.30E-11	0.007948	0.01195	0.5059
Smoking Index	PAD	rs4543592	T	C	0.52	-0.012	0.002	4.90E-10	-0.002519	0.01021	0.8052
Smoking Index	PAD	rs4571506	C	T	0.54	0.011	0.002	1.60E-08	0.006118	0.0104	0.5563
Smoking Index	PAD	rs4671357	T	C	0.519	-0.014	0.002	1.10E-11	-0.0209	0.01039	0.04428
Smoking Index	PAD	rs4814873	C	T	0.767	0.014	0.002	2.70E-09	0.01488	0.01205	0.2168
Smoking Index	PAD	rs4949465	T	C	0.87	-0.017	0.003	1.50E-08	-0.01876	0.01518	0.2171
Smoking Index	PAD	rs4957528	A	C	0.208	-0.015	0.002	4.50E-09	-0.000419	0.01329	0.9748
Smoking Index	PAD	rs549845	G	A	0.301	0.016	0.002	9.90E-14	0.01163	0.01094	0.2883
Smoking Index	PAD	rs6011779	C	T	0.191	0.028	0.003	1.60E-27	0.026	0.01272	0.04133
Smoking Index	PAD	rs60952428	T	C	0.909	0.019	0.003	3.40E-08	0.01718	0.0179	0.3363
Smoking Index	PAD	rs6119897	G	A	0.762	-0.018	0.002	3.10E-15	-0.003401	0.01185	0.7741
Smoking Index	PAD	rs62098013	G	A	0.64	-0.012	0.002	5.60E-09	-0.01932	0.01124	0.08583
Smoking Index	PAD	rs62135536	C	T	0.968	0.035	0.006	6.80E-10	0.01148	0.03157	0.7159
Smoking Index	PAD	rs62155874	A	G	0.873	-0.024	0.003	4.00E-16	-0.01355	0.01574	0.3898
Smoking Index	PAD	rs62175972	T	C	0.966	0.031	0.006	1.70E-08	0.01678	0.02923	0.5651

© 2021 Levin MG et al. *JAMA Network Open*.

Smoking Index	PAD	rs624833	T	G	0.695	0.013	0.002	9.20E-10	0.0309	0.01111	0.005333
Smoking Index	PAD	rs6598539	T	C	0.489	-0.012	0.002	3.70E-09	-0.02884	0.0102	0.004695
Smoking Index	PAD	rs6741228	T	C	0.433	0.011	0.002	1.70E-08	0.01758	0.01061	0.09752
Smoking Index	PAD	rs67596067	G	A	0.649	-0.013	0.002	1.10E-09	-0.01481	0.01074	0.168
Smoking Index	PAD	rs6778080	T	C	0.267	0.016	0.002	2.10E-12	0.004017	0.01135	0.7235
Smoking Index	PAD	rs6779302	G	T	0.633	-0.013	0.002	1.40E-09	-0.01217	0.0106	0.2513
Smoking Index	PAD	rs6935954	A	G	0.421	0.014	0.002	9.60E-12	-0.003373	0.01023	0.7416
Smoking Index	PAD	rs6957896	C	T	0.503	-0.011	0.002	1.90E-08	0.001631	0.01017	0.8726
Smoking Index	PAD	rs6962772	A	G	0.846	0.016	0.003	8.60E-09	-0.005086	0.01408	0.718
Smoking Index	PAD	rs7039819	G	A	0.427	0.013	0.002	5.20E-10	-0.006572	0.01052	0.5323
Smoking Index	PAD	rs7077678	C	T	0.623	0.012	0.002	2.40E-09	0.006004	0.01067	0.5737
Smoking Index	PAD	rs71367545	G	A	0.791	-0.015	0.002	1.70E-09	-0.01171	0.01261	0.3534
Smoking Index	PAD	rs7144406	A	G	0.791	-0.013	0.002	4.20E-08	-0.0006525	0.01273	0.9591
Smoking Index	PAD	rs7155595	A	C	0.674	-0.013	0.002	3.40E-09	-0.01944	0.01092	0.07521
Smoking Index	PAD	rs72678864	G	A	0.829	0.018	0.003	1.70E-11	0.02147	0.0141	0.1272
Smoking Index	PAD	rs7297175	T	C	0.431	-0.012	0.002	1.10E-08	0.005548	0.01024	0.5881
Smoking Index	PAD	rs732083	G	A	0.333	0.012	0.002	1.40E-08	0.01443	0.01081	0.1823
Smoking Index	PAD	rs73220544	A	C	0.842	-0.016	0.003	1.50E-08	-0.01451	0.01472	0.3248
Smoking Index	PAD	rs74086911	G	A	0.925	0.021	0.004	2.00E-08	0.05246	0.01975	0.007594
Smoking Index	PAD	rs7528604	G	A	0.566	0.014	0.002	6.60E-12	0.002456	0.01028	0.8112
Smoking Index	PAD	rs7553348	G	A	0.438	0.014	0.002	5.90E-12	0.009282	0.01035	0.3697
Smoking Index	PAD	rs7569203	A	C	0.689	-0.016	0.002	5.50E-13	-0.02465	0.01102	0.02543
Smoking Index	PAD	rs75742406	G	A	0.739	0.014	0.002	1.20E-09	-0.01245	0.0121	0.3042
Smoking Index	PAD	rs76608582	C	A	0.953	0.031	0.005	3.40E-10	0.01947	0.02406	0.4175
Smoking Index	PAD	rs7766610	C	A	0.183	0.018	0.003	2.40E-12	0.03032	0.01319	0.02173
Smoking Index	PAD	rs7775552	A	G	0.513	-1.20E-02	0.002	2.50E-09	-0.01445	0.01012	0.1533
Smoking Index	PAD	rs7807019	A	G	0.54	-0.015	0.002	7.50E-14	-0.02599	0.01026	0.01131
Smoking Index	PAD	rs8042849	C	T	0.342	2.80E-02	0.002	2.40E-39	0.05263	0.01072	9.38E-07

© 2021 Levin MG et al. *JAMA Network Open*.

Smoking Index	PAD	rs860326	C	T	0.428	0.012	0.002	3.20E-09	0.005334	0.01035	0.6064
Smoking Index	PAD	rs889398	C	T	0.588	0.013	0.002	3.90E-11	0.05439	0.01063	3.04E-07
Smoking Index	PAD	rs912780	T	G	0.65	0.012	0.002	8.50E-09	-0.004179	0.01069	0.696
Smoking Index	PAD	rs9842947	C	T	0.326	-0.012	0.002	4.40E-09	-0.01687	0.01141	0.1391
Smoking Index	PAD	rs986391	G	A	0.367	0.016	0.002	1.30E-14	0.02285	0.01052	0.02987
Smoking Index	PAD	rs9904288	T	C	0.708	0.012	0.002	2.40E-08	0.0318	0.0112	0.004475
Smoking Index	PAD	rs9919670	G	A	0.612	-0.022	0.002	2.10E-26	-0.0009182	0.01044	0.9299
Smoking Index	STROKE	rs10282292	C	T	0.362	0.013	0.002	7.10E-10	-0.0212	0.0208	0.3068
Smoking Index	STROKE	rs1050847	C	T	0.426	0.011	0.002	1.80E-08	0.0238	0.0245	0.3316
Smoking Index	STROKE	rs10516044	G	C	0.884	-0.02	0.003	2.00E-10	0.0011	0.0346	0.9754
Smoking Index	STROKE	rs10879871	T	G	0.343	-0.014	0.002	5.60E-11	-0.0024	0.0209	0.9078
Smoking Index	STROKE	rs10918701	G	A	0.372	0.012	0.002	2.00E-08	0.0127	0.0209	0.5439
Smoking Index	STROKE	rs111243290	T	C	0.963	-0.029	0.005	4.30E-08	-0.042	0.0586	0.4735
Smoking Index	STROKE	rs1118814	G	A	0.324	0.012	0.002	1.40E-08	0.0176	0.0214	0.4099
Smoking Index	STROKE	rs11210229	A	G	0.384	0.017	0.002	2.80E-16	-0.0036	0.0208	0.8638
Smoking Index	STROKE	rs112282219	G	A	0.959	-0.033	0.005	3.70E-11	-0.094	0.0674	0.1631
Smoking Index	STROKE	rs11255908	T	G	0.743	-0.015	0.002	1.90E-10	0.0203	0.023	0.3767
Smoking Index	STROKE	rs113382419	C	A	0.889	-0.041	0.003	9.70E-38	-0.0143	0.0444	0.7474
Smoking Index	STROKE	rs1149331	G	A	0.477	-0.011	0.002	3.20E-08	-0.0332	0.0221	0.1327
Smoking Index	STROKE	rs11768481	C	A	0.666	0.013	0.002	1.00E-09	-0.0331	0.0261	0.2044
Smoking Index	STROKE	rs11783093	C	T	0.839	0.023	0.003	1.00E-16	0.016	0.0332	0.6306

© 2021 Levin MG et al. *JAMA Network Open*.

Smoking Index	STROKE	rs11861214	G	T	0.784	0.013	0.002	2.60E-08	-0.0404	0.0233	0.0821997
Smoking Index	STROKE	rs11948770	T	C	0.768	-0.015	0.002	4.00E-10	0.007	0.0242	0.7718
Smoking Index	STROKE	rs1221148	C	G	0.587	0.013	0.002	4.60E-11	-0.0311	0.0204	0.1271
Smoking Index	STROKE	rs12244388	G	A	0.661	-0.019	0.002	9.60E-20	0.0254	0.0201	0.2059
Smoking Index	STROKE	rs1246265	T	C	0.305	-0.013	0.002	5.30E-09	-0.0432	0.0222	0.0516904
Smoking Index	STROKE	rs12481282	G	C	0.722	-0.013	0.002	7.80E-09	-0.0071	0.0221	0.75
Smoking Index	STROKE	rs12623702	A	G	0.613	-0.014	0.002	5.70E-12	0.0494	0.0213	0.0203498
Smoking Index	STROKE	rs12708665	A	G	0.285	-0.013	0.002	3.40E-09	-0.0022	0.0244	0.9292
Smoking Index	STROKE	rs12831617	C	T	0.764	-0.013	0.002	2.40E-08	-0.037	0.026	0.1548
Smoking Index	STROKE	rs12967855	A	G	0.331	0.012	0.002	4.00E-08	-0.007	0.0219	0.7485
Smoking Index	STROKE	rs13009008	A	G	0.328	0.012	0.002	6.40E-09	-0.0029	0.0212	0.8921
Smoking Index	STROKE	rs13016665	C	A	0.577	-0.012	0.002	1.40E-09	-0.0572	0.0203	0.00487697
Smoking Index	STROKE	rs13296519	G	T	0.606	-0.014	0.002	1.40E-11	-0.0079	0.0223	0.725
Smoking Index	STROKE	rs136233	A	G	0.809	-0.014	0.003	2.70E-08	0.04	0.0269	0.1369
Smoking Index	STROKE	rs147412694	G	A	0.85	-0.017	0.003	2.30E-09	0.0307	0.0341	0.3687
Smoking Index	STROKE	rs17309874	G	A	0.74	-0.016	0.002	5.60E-13	-0.0015	0.0281	0.9568
Smoking Index	STROKE	rs17553262	A	C	0.885	-0.018	0.003	6.90E-09	-0.0313	0.0334	0.3484
Smoking Index	STROKE	rs17576594	G	A	0.724	0.016	0.002	1.90E-12	-0.01	0.0227	0.6607

© 2021 Levin MG et al. *JAMA Network Open*.

Smoking Index	STROKE	rs1827535	T	G	0.686	-0.012	0.002	1.20E-08	0.0081	0.0251	0.7462
Smoking Index	STROKE	rs1922018	C	T	0.364	0.014	0.002	2.50E-12	-0.0176	0.0217	0.4157
Smoking Index	STROKE	rs1931263	G	T	0.51	-0.011	0.002	4.00E-08	0.0353	0.0232	0.1277
Smoking Index	STROKE	rs1933270	T	G	0.364	0.013	0.002	1.70E-10	0.0225	0.0208	0.2781
Smoking Index	STROKE	rs202645	A	G	0.203	-0.015	0.002	5.00E-09	0.0144	0.0278	0.6052
Smoking Index	STROKE	rs2062882	G	A	0.587	-0.012	0.002	1.00E-08	0.0432	0.021	0.0397201
Smoking Index	STROKE	rs2080870	A	T	0.258	0.012	0.002	4.50E-08	-0.0263	0.0265	0.3219
Smoking Index	STROKE	rs215600	G	A	0.358	0.017	0.002	2.00E-15	0.0142	0.0207	0.4932
Smoking Index	STROKE	rs2160896	C	T	0.317	-0.012	0.002	2.20E-08	0.0191	0.021	0.365
Smoking Index	STROKE	rs2254710	C	A	0.236	0.013	0.002	3.00E-08	0.0251	0.0229	0.272
Smoking Index	STROKE	rs245774	A	G	0.272	-0.013	0.002	9.00E-09	0.0046	0.0248	0.8523
Smoking Index	STROKE	rs2675638	G	A	0.581	0.012	0.002	1.20E-09	0.0289	0.0205	0.1573
Smoking Index	STROKE	rs2838834	C	T	0.699	-0.013	0.002	9.20E-10	0.0098	0.0221	0.6584
Smoking Index	STROKE	rs28485305	C	T	0.631	0.011	0.002	2.80E-08	0.0354	0.0205	0.0840098
Smoking Index	STROKE	rs2867112	T	G	0.835	0.021	0.003	8.30E-15	0.0123	0.0281	0.662599
Smoking Index	STROKE	rs2890772	G	T	0.413	-0.02	0.002	3.60E-22	-0.0104	0.0206	0.6132
Smoking Index	STROKE	rs2894808	T	A	0.922	-0.022	0.004	4.50E-09	-0.0155	0.0381	0.6831
Smoking Index	STROKE	rs317021	T	A	0.814	-0.017	0.003	6.80E-11	-0.0474	0.0328	0.148

© 2021 Levin MG et al. *JAMA Network Open*.

Smoking Index	STROKE	rs326341	G	A	0.525	0.014	0.002	8.70E-12	0.023	0.0201	0.2525
Smoking Index	STROKE	rs329120	C	T	0.581	0.014	0.002	5.80E-12	-0.0105	0.0199	0.5975
Smoking Index	STROKE	rs348809	A	G	0.348	-0.012	0.002	1.90E-08	0.0187	0.0215	0.3856
Smoking Index	STROKE	rs35120974	A	G	0.583	-0.012	0.002	2.10E-09	-0.03	0.0204	0.1415
Smoking Index	STROKE	rs35169606	T	G	0.612	0.013	0.002	1.20E-09	0.0426	0.0248	0.0860796
Smoking Index	STROKE	rs35175834	G	A	0.788	-0.024	0.002	4.80E-22	-0.0096	0.0274	0.7259
Smoking Index	STROKE	rs35343344	C	A	0.733	0.013	0.002	1.00E-08	0.0108	0.0238	0.6511
Smoking Index	STROKE	rs359243	T	C	0.393	-0.013	0.002	8.30E-10	-0.0043	0.0222	0.8453
Smoking Index	STROKE	rs369230	G	T	0.308	-0.013	0.002	2.00E-09	0.0347	0.0237	0.1434
Smoking Index	STROKE	rs3742365	T	C	0.595	-0.016	0.002	2.30E-14	-0.0012	0.0203	0.9539
Smoking Index	STROKE	rs3811038	T	C	0.724	-0.014	0.002	9.20E-10	0.011	0.0242	0.649999
Smoking Index	STROKE	rs3896224	A	G	0.585	0.014	0.002	1.00E-11	-0.0021	0.0203	0.9177
Smoking Index	STROKE	rs421983	T	C	0.519	0.013	0.002	3.10E-10	0.0095	0.02	0.6351
Smoking Index	STROKE	rs4391802	A	G	0.707	0.015	0.002	1.30E-11	0.0467	0.0228	0.0405098
Smoking Index	STROKE	rs4473348	A	T	0.25	-0.015	0.002	9.00E-11	0.0351	0.0235	0.135
Smoking Index	STROKE	rs4543592	T	C	0.52	-0.012	0.002	4.90E-10	0.0268	0.023	0.2449
Smoking Index	STROKE	rs4571506	C	T	0.54	0.011	0.002	1.60E-08	0.01	0.0199	0.614601
Smoking Index	STROKE	rs4671357	T	C	0.519	-0.014	0.002	1.10E-11	-0.0065	0.02	0.743901

© 2021 Levin MG et al. *JAMA Network Open*.

Smoking Index	STROKE	rs4814873	C	T	0.767	0.014	0.002	2.70E-09	0.0296	0.0239	0.2157
Smoking Index	STROKE	rs4949465	T	C	0.87	-0.017	0.003	1.50E-08	0.0166	0.0282	0.5562
Smoking Index	STROKE	rs4957528	A	C	0.208	-0.015	0.002	4.50E-09	0.0042	0.0298	0.8883
Smoking Index	STROKE	rs549845	G	A	0.301	0.016	0.002	9.90E-14	0.0415	0.0212	0.0503999
Smoking Index	STROKE	rs6011779	C	T	0.191	0.028	0.003	1.60E-27	0.0023	0.0288	0.9365
Smoking Index	STROKE	rs60952428	T	C	0.909	0.019	0.003	3.40E-08	0.0086	0.029	0.7663
Smoking Index	STROKE	rs6119897	G	A	0.762	-0.018	0.002	3.10E-15	-0.0001	0.0238	0.9968
Smoking Index	STROKE	rs62098013	G	A	0.64	-0.012	0.002	5.60E-09	0.0101	0.0246	0.6806
Smoking Index	STROKE	rs62155874	A	G	0.873	-0.024	0.003	4.00E-16	0.0044	0.0354	0.9009
Smoking Index	STROKE	rs62175972	T	C	0.966	0.031	0.006	1.70E-08	0.0949	0.0807	0.2399
Smoking Index	STROKE	rs624833	T	G	0.695	0.013	0.002	9.20E-10	-0.0012	0.0251	0.9629
Smoking Index	STROKE	rs6598539	T	C	0.489	-0.012	0.002	3.70E-09	0.0078	0.0203	0.701601
Smoking Index	STROKE	rs6741228	T	C	0.433	0.011	0.002	1.70E-08	0.0185	0.0204	0.3656
Smoking Index	STROKE	rs67596067	G	A	0.649	-0.013	0.002	1.10E-09	-0.0215	0.022	0.328
Smoking Index	STROKE	rs6778080	T	C	0.267	0.016	0.002	2.10E-12	-0.0511	0.0278	0.0665197
Smoking Index	STROKE	rs6935954	A	G	0.421	0.014	0.002	9.60E-12	-0.0487	0.0232	0.0361901
Smoking Index	STROKE	rs6957896	C	T	0.503	-0.011	0.002	1.90E-08	0.0018	0.0226	0.9351
Smoking Index	STROKE	rs6962772	A	G	0.846	0.016	0.003	8.60E-09	0.0363	0.0266	0.173

© 2021 Levin MG et al. *JAMA Network Open*.

Smoking Index	STROKE	rs7039819	G	A	0.427	0.013	0.002	5.20E-10	0.0098	0.0206	0.632501
Smoking Index	STROKE	rs7077678	C	T	0.623	0.012	0.002	2.40E-09	-0.0056	0.0205	0.785301
Smoking Index	STROKE	rs71367545	G	A	0.791	-0.015	0.002	1.70E-09	-0.0394	0.0285	0.167
Smoking Index	STROKE	rs7144406	A	G	0.791	-0.013	0.002	4.20E-08	-0.025	0.0307	0.4156
Smoking Index	STROKE	rs7155595	A	C	0.674	-0.013	0.002	3.40E-09	-0.0549	0.0237	0.0203002
Smoking Index	STROKE	rs72678864	G	A	0.829	0.018	0.003	1.70E-11	0.0311	0.0337	0.356
Smoking Index	STROKE	rs7297175	T	C	0.431	-0.012	0.002	1.10E-08	-0.0247	0.0215	0.2517
Smoking Index	STROKE	rs732083	G	A	0.333	0.012	0.002	1.40E-08	0.0258	0.0214	0.2281
Smoking Index	STROKE	rs73220544	A	C	0.842	-0.016	0.003	1.50E-08	-0.0699	0.0289	0.0157601
Smoking Index	STROKE	rs7333559	G	A	0.212	0.015	0.002	5.30E-10	-0.0158	0.0253	0.531401
Smoking Index	STROKE	rs74086911	G	A	0.925	0.021	0.004	2.00E-08	0.0525	0.043	0.222
Smoking Index	STROKE	rs7528604	G	A	0.566	0.014	0.002	6.60E-12	0.0229	0.0201	0.2533
Smoking Index	STROKE	rs7553348	G	A	0.438	0.014	0.002	5.90E-12	-0.0052	0.0228	0.8208
Smoking Index	STROKE	rs7569203	A	C	0.689	-0.016	0.002	5.50E-13	0.0062	0.0216	0.7732
Smoking Index	STROKE	rs75742406	G	A	0.739	0.014	0.002	1.20E-09	-0.0381	0.0224	0.089041
Smoking Index	STROKE	rs76608582	C	A	0.953	0.031	0.005	3.40E-10	0.0348	0.0801	0.664
Smoking Index	STROKE	rs7766610	C	A	0.183	0.018	0.003	2.40E-12	-0.0133	0.0233	0.568
Smoking Index	STROKE	rs7775552	A	G	0.513	-0.012	0.002	2.50E-09	-0.0204	0.0204	0.317

© 2021 Levin MG et al. *JAMA Network Open*.

Smoking Index	STROKE	rs7807019	A	G	0.54	-0.015	0.002	7.50E-14	0.0001	0.0201	0.9977
Smoking Index	STROKE	rs8042134	T	G	0.541	-0.014	0.002	2.20E-12	0.0438	0.0212	0.0389798
Smoking Index	STROKE	rs8042849	C	T	0.342	0.028	0.002	2.40E-39	0.0173	0.0223	0.4364
Smoking Index	STROKE	rs860326	C	T	0.428	0.012	0.002	3.20E-09	0.0142	0.021	0.4971
Smoking Index	STROKE	rs8614	C	A	0.818	-0.017	0.003	1.50E-10	-0.0106	0.024	0.6592
Smoking Index	STROKE	rs889398	C	T	0.588	0.013	0.002	3.90E-11	0.0454	0.0217	0.0368002
Smoking Index	STROKE	rs912780	T	G	0.65	0.012	0.002	8.50E-09	0.0349	0.0205	0.088369
Smoking Index	STROKE	rs9435340	T	A	0.344	0.012	0.002	1.00E-08	0.0069	0.0212	0.7438
Smoking Index	STROKE	rs9842947	C	T	0.326	-0.012	0.002	4.40E-09	-0.0075	0.0223	0.7364
Smoking Index	STROKE	rs986391	G	A	0.367	0.016	0.002	1.30E-14	0.0057	0.0215	0.7904
Smoking Index	STROKE	rs9904288	T	C	0.708	0.012	0.002	2.40E-08	0.0335	0.0226	0.1393
Smoking Index	STROKE	rs9919670	G	A	0.612	-0.022	0.002	2.10E-26	-0.0378	0.0213	0.0762799
Smoking Index	CAD	rs10282292	C	T	0.362	0.013	0.002	7.10E-10	0.022719	0.0095582	0.0174578
Smoking Index	CAD	rs10879871	T	G	0.343	-0.014	0.002	5.60E-11	-0.002138	0.0094615	0.821226
Smoking Index	CAD	rs10918701	G	A	0.372	0.012	0.002	2.00E-08	0.023604	0.0095679	0.0136254
Smoking Index	CAD	rs111243290	T	C	0.963	-0.029	0.005	4.30E-08	0.00071	0.0282052	0.979917
Smoking Index	CAD	rs1118814	G	A	0.324	0.012	0.002	1.40E-08	-0.003204	0.0098265	0.744381
Smoking Index	CAD	rs11210229	A	G	0.384	0.017	0.002	2.80E-16	0.000119	0.0094202	0.989921

© 2021 Levin MG et al. *JAMA Network Open*.

Smoking Index	CAD	rs112282219	G	A	0.959	-0.033	0.005	3.70E-11	0.001997	0.0273155	0.94172
Smoking Index	CAD	rs11255908	T	G	0.743	-0.015	0.002	1.90E-10	0.010164	0.0102002	0.319029
Smoking Index	CAD	rs113382419	C	A	0.889	-0.041	0.003	9.70E-38	-0.02074	0.0189892	0.274746
Smoking Index	CAD	rs1149331	G	A	0.477	-0.011	0.002	3.20E-08	-0.007664	0.0094702	0.418355
Smoking Index	CAD	rs11768481	C	A	0.666	0.013	0.002	1.00E-09	0.014147	0.010438	0.175312
Smoking Index	CAD	rs11783093	C	T	0.839	0.023	0.003	1.00E-16	0.034962	0.0138305	0.0114749
Smoking Index	CAD	rs11861214	G	T	0.784	0.013	0.002	2.60E-08	-0.002208	0.0111952	0.843649
Smoking Index	CAD	rs11948770	T	C	0.768	-0.015	0.002	4.00E-10	0.005828	0.011319	0.606633
Smoking Index	CAD	rs1221148	C	G	0.587	0.013	0.002	4.60E-11	-0.000828	0.0094973	0.930526
Smoking Index	CAD	rs12244388	G	A	0.661	-0.019	0.002	9.60E-20	0.036276	0.0094343	0.000120501
Smoking Index	CAD	rs1246265	T	C	0.305	-0.013	0.002	5.30E-09	-0.009105	0.0101448	0.369448
Smoking Index	CAD	rs12481282	G	C	0.722	-0.013	0.002	7.80E-09	0.026601	0.0103881	0.0104455
Smoking Index	CAD	rs12623702	A	G	0.613	-0.014	0.002	5.70E-12	0.008648	0.0099101	0.382857
Smoking Index	CAD	rs12708665	A	G	0.285	-0.013	0.002	3.40E-09	-0.010716	0.0106944	0.316334
Smoking Index	CAD	rs12831617	C	T	0.764	-0.013	0.002	2.40E-08	-0.023783	0.0114537	0.037853
Smoking Index	CAD	rs12967855	A	G	0.331	0.012	0.002	4.00E-08	-0.009557	0.0097124	0.325116
Smoking Index	CAD	rs13009008	A	G	0.328	0.012	0.002	6.40E-09	0.003304	0.0101295	0.744291
Smoking Index	CAD	rs13016665	C	A	0.577	-0.012	0.002	1.40E-09	-0.005148	0.0097971	0.599264

© 2021 Levin MG et al. *JAMA Network Open*.

Smoking Index	CAD	rs136233	A	G	0.809	-0.014	0.003	2.70E-08	-0.030685	0.0119425	0.0101878
Smoking Index	CAD	rs147412694	G	A	0.85	-0.017	0.003	2.30E-09	-0.014472	0.0141303	0.30575
Smoking Index	CAD	rs17309874	G	A	0.74	-0.016	0.002	5.60E-13	-0.024627	0.0117992	0.0368714
Smoking Index	CAD	rs17553262	A	C	0.885	-0.018	0.003	6.90E-09	0.004595	0.0165375	0.781124
Smoking Index	CAD	rs17576594	G	A	0.724	0.016	0.002	1.90E-12	-0.001252	0.0102642	0.902917
Smoking Index	CAD	rs1827535	T	G	0.686	-0.012	0.002	1.20E-08	-0.013067	0.0105988	0.217623
Smoking Index	CAD	rs1922018	C	T	0.364	0.014	0.002	2.50E-12	-0.006448	0.010022	0.519974
Smoking Index	CAD	rs1931263	G	T	0.51	-0.011	0.002	4.00E-08	-0.013821	0.0097993	0.15842
Smoking Index	CAD	rs202645	A	G	0.203	-0.015	0.002	5.00E-09	-0.00722	0.0112549	0.5212
Smoking Index	CAD	rs2062882	G	A	0.587	-0.012	0.002	1.00E-08	-0.000649	0.0095049	0.945562
Smoking Index	CAD	rs2080870	A	T	0.258	0.012	0.002	4.50E-08	-0.014601	0.0111898	0.191942
Smoking Index	CAD	rs215600	G	A	0.358	0.017	0.002	2.00E-15	0.014977	0.0095495	0.116797
Smoking Index	CAD	rs2160896	C	T	0.317	-0.012	0.002	2.20E-08	-0.01444	0.0097656	0.139233
Smoking Index	CAD	rs2254710	C	A	0.236	0.013	0.002	3.00E-08	0.019446	0.0104685	0.0632281
Smoking Index	CAD	rs245774	A	G	0.272	-0.013	0.002	9.00E-09	-0.011289	0.0112452	0.315427
Smoking Index	CAD	rs2675638	G	A	0.581	0.012	0.002	1.20E-09	0.006059	0.0092768	0.51367
Smoking Index	CAD	rs2838834	C	T	0.699	-0.013	0.002	9.20E-10	-0.021708	0.009846	0.027472
Smoking Index	CAD	rs2867112	T	G	0.835	0.021	0.003	8.30E-15	0.041434	0.0125237	0.000938102
Smoking Index	CAD	rs2890772	G	T	0.413	-0.02	0.002	3.60E-22	0.003933	0.0097363	0.686248

© 2021 Levin MG et al. *JAMA Network Open*.

Smoking Index	CAD	rs2894808	T	A	0.922	-0.022	0.004	4.50E-09	-0.004522	0.0166581	0.786037
Smoking Index	CAD	rs317021	T	A	0.814	-0.017	0.003	6.80E-11	-0.000821	0.012704	0.948472
Smoking Index	CAD	rs326341	G	A	0.525	0.014	0.002	8.70E-12	-0.003695	0.0093626	0.693099
Smoking Index	CAD	rs329120	C	T	0.581	0.014	0.002	5.80E-12	-0.020066	0.0093474	0.0318178
Smoking Index	CAD	rs348809	A	G	0.348	-0.012	0.002	1.90E-08	-0.006893	0.0097309	0.478719
Smoking Index	CAD	rs35120974	A	G	0.583	-0.012	0.002	2.10E-09	0.001829	0.0093865	0.845507
Smoking Index	CAD	rs35169606	T	G	0.612	0.013	0.002	1.20E-09	0.012571	0.011193	0.26139
Smoking Index	CAD	rs35175834	G	A	0.788	-0.024	0.002	4.80E-22	0.011184	0.0120284	0.352473
Smoking Index	CAD	rs35343344	C	A	0.733	0.013	0.002	1.00E-08	0.01511	0.0112408	0.17888
Smoking Index	CAD	rs3742365	T	C	0.595	-0.016	0.002	2.30E-14	-0.016205	0.0094984	0.0879934
Smoking Index	CAD	rs3811038	T	C	0.724	-0.014	0.002	9.20E-10	0.004533	0.0109681	0.679396
Smoking Index	CAD	rs421983	T	C	0.519	0.013	0.002	3.10E-10	0.00063	0.0091541	0.945131
Smoking Index	CAD	rs4391802	A	G	0.707	0.015	0.002	1.30E-11	0.004798	0.0102219	0.638794
Smoking Index	CAD	rs4473348	A	T	0.25	-0.015	0.002	9.00E-11	-0.020153	0.0110681	0.0686341
Smoking Index	CAD	rs4671357	T	C	0.519	-0.014	0.002	1.10E-11	-0.008261	0.0094528	0.382161
Smoking Index	CAD	rs4814873	C	T	0.767	0.014	0.002	2.70E-09	0.021137	0.0109073	0.0526393
Smoking Index	CAD	rs4949465	T	C	0.87	-0.017	0.003	1.50E-08	-0.022869	0.0122141	0.0611589
Smoking Index	CAD	rs4957528	A	C	0.208	-0.015	0.002	4.50E-09	-0.014558	0.0127464	0.253401
Smoking Index	CAD	rs549845	G	A	0.301	0.016	0.002	9.90E-14	0.008157	0.0098276	0.406531

© 2021 Levin MG et al. *JAMA Network Open*.

Smoking Index	CAD	rs6011779	C	T	0.191	0.028	0.003	1.60E-27	0.018733	0.0135459	0.166687
Smoking Index	CAD	rs60952428	T	C	0.909	0.019	0.003	3.40E-08	0.014414	0.0138815	0.299104
Smoking Index	CAD	rs6119897	G	A	0.762	-0.018	0.002	3.10E-15	-0.020157	0.0105173	0.0552955
Smoking Index	CAD	rs62098013	G	A	0.64	-0.012	0.002	5.60E-09	-0.008057	0.0106662	0.450021
Smoking Index	CAD	rs62135536	C	T	0.968	0.035	0.006	6.80E-10	0.003779	0.0298238	0.899169
Smoking Index	CAD	rs62155874	A	G	0.873	-0.024	0.003	4.00E-16	0.017265	0.0153904	0.261946
Smoking Index	CAD	rs62175972	T	C	0.966	0.031	0.006	1.70E-08	0.030329	0.0304353	0.319005
Smoking Index	CAD	rs624833	T	G	0.695	0.013	0.002	9.20E-10	0.010431	0.0106792	0.328689
Smoking Index	CAD	rs6741228	T	C	0.433	0.011	0.002	1.70E-08	0.020156	0.0093409	0.0309421
Smoking Index	CAD	rs67596067	G	A	0.649	-0.013	0.002	1.10E-09	-0.017072	0.009959	0.0864868
Smoking Index	CAD	rs6778080	T	C	0.267	0.016	0.002	2.10E-12	0.0106	0.0114407	0.354177
Smoking Index	CAD	rs6779302	G	T	0.633	-0.013	0.002	1.40E-09	0.002586	0.0097917	0.791702
Smoking Index	CAD	rs6935954	A	G	0.421	0.014	0.002	9.60E-12	-0.010899	0.0098916	0.270532
Smoking Index	CAD	rs6957896	C	T	0.503	-0.011	0.002	1.90E-08	-0.00727	0.0095516	0.446582
Smoking Index	CAD	rs6962772	A	G	0.846	0.016	0.003	8.60E-09	0.008701	0.0114472	0.447196
Smoking Index	CAD	rs7039819	G	A	0.427	0.013	0.002	5.20E-10	0.014408	0.0094865	0.128814
Smoking Index	CAD	rs7077678	C	T	0.623	0.012	0.002	2.40E-09	0.011614	0.0093355	0.213473
Smoking Index	CAD	rs71367545	G	A	0.791	-0.015	0.002	1.70E-09	0.003924	0.0122022	0.74777

© 2021 Levin MG et al. *JAMA Network Open*.

Smoking Index	CAD	rs7144406	A	G	0.791	-0.013	0.002	4.20E-08	-0.003584	0.0129962	0.782723
Smoking Index	CAD	rs7155595	A	C	0.674	-0.013	0.002	3.40E-09	-0.008617	0.0102653	0.40123
Smoking Index	CAD	rs72678864	G	A	0.829	0.018	0.003	1.70E-11	0.010335	0.014001	0.460418
Smoking Index	CAD	rs7297175	T	C	0.431	-0.012	0.002	1.10E-08	0.004107	0.0098308	0.676117
Smoking Index	CAD	rs732083	G	A	0.333	0.012	0.002	1.40E-08	0.020676	0.0098276	0.0353891
Smoking Index	CAD	rs73220544	A	C	0.842	-0.016	0.003	1.50E-08	-0.008214	0.0133113	0.537188
Smoking Index	CAD	rs7333559	G	A	0.212	0.015	0.002	5.30E-10	0.00524	0.0120327	0.663214
Smoking Index	CAD	rs74086911	G	A	0.925	0.021	0.004	2.00E-08	0.001896	0.0189697	0.920385
Smoking Index	CAD	rs7528604	G	A	0.566	0.014	0.002	6.60E-12	0.010029	0.0093105	0.281403
Smoking Index	CAD	rs7553348	G	A	0.438	0.014	0.002	5.90E-12	-0.003328	0.00943	0.724151
Smoking Index	CAD	rs7569203	A	C	0.689	-0.016	0.002	5.50E-13	0.000949	0.0098428	0.92319
Smoking Index	CAD	rs75742406	G	A	0.739	0.014	0.002	1.20E-09	0.008049	0.0105923	0.44732
Smoking Index	CAD	rs76608582	C	A	0.953	0.031	0.005	3.40E-10	-0.03298	0.0379491	0.384815
Smoking Index	CAD	rs7766610	C	A	0.183	0.018	0.003	2.40E-12	0.012158	0.0109866	0.268458
Smoking Index	CAD	rs7775552	A	G	0.513	-0.012	0.002	2.50E-09	-0.002453	0.0092801	0.791526
Smoking Index	CAD	rs7807019	A	G	0.54	-0.015	0.002	7.50E-14	-0.018949	0.0091877	0.0391679
Smoking Index	CAD	rs8042134	T	G	0.541	-0.014	0.002	2.20E-12	0.013937	0.0097048	0.150976
Smoking Index	CAD	rs8042849	C	T	0.342	0.028	0.002	2.40E-39	-0.003889	0.0101056	0.700359
Smoking Index	CAD	rs860326	C	T	0.428	0.012	0.002	3.20E-09	0.007768	0.009569	0.416915

© 2021 Levin MG et al. *JAMA Network Open*.

Smoking Index	CAD	rs8614	C	A	0.818	-0.017	0.003	1.50E-10	-0.021264	0.0116731	0.0685125
Smoking Index	CAD	rs889398	C	T	0.588	0.013	0.002	3.90E-11	0.026764	0.0097081	0.00583553
Smoking Index	CAD	rs912780	T	G	0.65	0.012	0.002	8.50E-09	0.004244	0.0095408	0.656444
Smoking Index	CAD	rs9435340	T	A	0.344	0.012	0.002	1.00E-08	0.011075	0.0097276	0.254907
Smoking Index	CAD	rs9842947	C	T	0.326	-0.012	0.002	4.40E-09	-0.000872	0.0099003	0.929815
Smoking Index	CAD	rs986391	G	A	0.367	0.016	0.002	1.30E-14	-0.000997	0.0095451	0.916811
Smoking Index	CAD	rs9904288	T	C	0.708	0.012	0.002	2.40E-08	0.045469	0.0103338	1.07999E-05
Smoking Index	CAD	rs9919670	G	A	0.612	-0.022	0.002	2.10E-26	0.007256	0.0094604	0.443089

© 2021 Levin MG et al. *JAMA Network Open*.

eTable 2. Egger Bias Intercept Test for Smoking Index-ASCVD Analysis

Exposure	Outcome	Egger Intercept	SE	P value
Smoking Index	PAD	-0.0077	0.0052	0.14
Smoking Index	STROKE	-0.0024	0.01	0.81
Smoking Index	CAD	0.01	0.0052	0.046
Smoking Initiation	PAD	-0.002	0.0077	0.79
Smoking Initiation	STROKE	-0.011	0.012	0.35
Smoking Initiation	CAD	-0.0031	0.0068	0.65

The Egger bias intercept test was applied to detect evidence of horizontal pleiotropy in the association between smoking and ASCVD outcomes. The test was applied using both exposures for genetic liability to smoking (lifetime smoking index and smoking initiation). P-values < 0.05 were considered evidence of potential horizontal pleiotropy.

© 2021 Levin MG et al. *JAMA Network Open*.

eTable 3. MR Steiger Directionality Test for Smoking-Cardiometabolic Risk Factor Analysis

Exposure	Outcome	r2 exposure	r2 outcome	Correct causal direction	Steiger pvalue
Smoking Index	Type 2 diabetes	0.01	0.0014	TRUE	3.60E-49
Smoking Index	Chronic kidney disease	0.0089	0.0012	TRUE	4.70E-73
Smoking Index	Overweight	0.0089	0.0012	TRUE	4.60E-90
Smoking Index	Hypertension	0.012	0.0017	TRUE	2.70E-236
Smoking Index	Hyperlipidemia	0.011	0.0016	TRUE	3.40E-224
Smoking Index	eGFR (creatinine)	0.0088	0.001	TRUE	1.70E-88
Smoking Index	Body mass index	0.009	0.0023	TRUE	1.20E-79
Smoking Index	HDL cholesterol	0.0089	0.0013	TRUE	3.00E-63
Smoking Index	LDL cholesterol	0.0089	0.001	TRUE	1.20E-70
Smoking Index	Total cholesterol	0.0089	0.0012	TRUE	1.90E-68
Smoking Index	Triglycerides	0.0089	0.0014	TRUE	4.50E-59
Smoking Index	Waist circumference	0.009	0.0026	TRUE	5.80E-52
Smoking Index	Waist-to-hip ratio	0.009	0.0016	TRUE	2.70E-75
Smoking Index	HbA1C	0.0089	0.0019	TRUE	6.60E-25
Smoking Index	Fasting glucose	0.0091	0.002	TRUE	3.60E-31
Smoking Index	Fasting insulin	0.0091	0.0023	TRUE	3.40E-25
Smoking Index	Interleukin-1 beta	0.012	0.046	FALSE	6.00E-10
Smoking Index	Interleukin-6	0.012	0.035	FALSE	0.0000045
Smoking Index	C-reactive protein	0.012	0.037	FALSE	0.0000014

© 2021 Levin MG et al. *JAMA Network Open*.

Smoking Index	Interleukin-6 receptor	0.0097	0.034	FALSE	3.60E-07
Smoking Index	Systolic Blood Pressure	0.012	0.0015	TRUE	3.10E-248

The MR Steiger directionality test was applied to determine the correct direction of effect for the estimated associations between smoking and cardiometabolic risk factors for ASCV.

© 2021 Levin MG et al. *JAMA Network Open*.
